# Prioritization and Sensitivity of Pesticide Risks from Root and Tuber Vegetables

**DOI:** 10.3390/jox15040125

**Published:** 2025-08-03

**Authors:** Milica Lučić, Antonije Onjia

**Affiliations:** 1 Innovation Center of the Faculty of Technology and Metallurgy, 11120 Belgrade, Serbia; milica.lucic@tmf.bg.ac.rs; 2 Department of Analytical Chemistry and Quality Control, Faculty of Technology and Metallurgy, University of Belgrade, 11120 Belgrade, Serbia

**Keywords:** potato, carrot, ginger, celery, onion, radish, QuEChERS, GC-MS/MS, HPLC-MS/MS, risk ranking, RASFF

## Abstract

This study investigated pesticide residues in 580 vegetable samples collected from markets in Serbia, encompassing potatoes, carrots, celery, radishes, horseradish, ginger, onions, and leeks. In total, 33 distinct pesticides were detected using validated HPLC-MS/MS and GC-MS/MS analytical methods. Multiple residues were identified in 19 samples, while 29 samples exceeded established maximum residue levels (MRLs). Acute and chronic dietary risks were assessed for both adults and children. Although individual hazard quotients (HQs) for adults and children remained below the threshold of concern (HQ < 1), the cumulative acute risk reached up to 63.1% of the Acute Reference Dose (ARfD) for children and 51.1% ARfD for adults, with ginger and celery posing the highest risks. Similarly, cumulative chronic risks remained below the safety threshold, with the Acceptable Daily Intake (ADI) percentages reaching a maximum of 5.9% ADI for adults and increased vulnerability of 11.0% ADI among children. Monte Carlo simulations were applied to account for variability and uncertainty in chronic exposure estimates. The hazard index (HI) results showed that adverse health effects for both population groups remained within acceptable safety limits (HI < 1), although higher susceptibility was observed in children. Sensitivity analysis identified body weight and vegetable consumption rates as the most influential factors affecting chronic risk variability.

## 1. Introduction

Pesticides are widely used in agriculture to control pests and plant diseases. These substances comprise a broad class of chemicals intentionally introduced into the environment to control weeds (herbicides), insect infestations (insecticides), fungal infections (fungicides), and rodents (rodenticides) [1,2]. In addition to their primary role in agriculture, pesticides are employed post-harvest to preserve crops and in non-agricultural settings such as residential, commercial, and public areas. However, the predominant application remains in agriculture, where the intensification of food production has inevitably led to increased pesticide use [1,3]. Due to their inherent toxicity, pesticides pose significant risks not only to human health but also to a wide range of non-target organisms. The adverse effects of pesticide exposure can be both acute and chronic, depending on the exposure dose and route [4]. Residual pesticide contamination in crops is a major concern because of their environmental persistence, potential for bioaccumulation, and toxicological impact. Upon release into the environment, pesticides may undergo degradation through abiotic processes or undergo transformation processes in organisms, yet their intermediate metabolites can also contribute to ecological and health risks [5,6].

Chronic exposure to pesticides has been associated with an increased risk of various health disorders, including cancer [7], endocrine disruption, cardiovascular diseases [8], respiratory conditions [9], and allergic reactions [10]. Moreover, prolonged or repeated pesticide exposure may negatively affect reproductive health, contributing to infertility and an elevated risk of miscarriage [11].

Despite the potential risks of pesticide contamination, vegetables remain an essential part of a healthy diet due to their high nutritional value. Vegetables are a fundamental component of a balanced diet, with regular consumption contributing positively to overall human health. Based on the edible plant part, vegetables are generally categorized into leafy, fruiting, root, tuber, and bulb vegetables [12]. Among root vegetables commonly included in the daily diet are carrot, celery, radish, horseradish, and parsley root. Potatoes are the most widely consumed tuber vegetable and rank as the third most produced food crop globally, following wheat and rice [13], and also serve as an important source of carbohydrates, dietary fiber, potassium, vitamin C, and other essential nutrients. Other frequently consumed vegetables, such as carrots, celery, onions, and ginger, are important sources of dietary fiber, vitamins, minerals, and various phytochemicals beneficial to human health [14,15,16,17,18].

Due to the widespread use of pesticides in vegetable cultivation, there is a growing need for strict control measures and limitations on excessive pesticide use [4]. Vegetables, such as potatoes, carrots, radishes, and celery, are often treated with herbicides, insecticides, and fungicides throughout the growing season to ensure yield quality and minimize post-harvest losses [2,4,6,19,20]. Because of their underground growth, roots, tubers, and bulb vegetables are particularly prone to pesticide accumulation, raising concerns about residue levels and the potential health risks associated with their consumption [12,21,22].

Maximum residue limits (MRLs), representing the highest concentrations of pesticide residues legally permitted in food or feed, are established by the European Commission under specific regulations [23]. These thresholds are derived from data obtained through Good Agricultural Practices (GAPs) and are essential criteria in the pesticide approval and registration process. Monitoring pesticide residues in vegetables is of critical importance, given the potential health risks associated with dietary exposure to these compounds [4,24]. Deterministic and probabilistic risk assessment approaches are commonly applied to assess the potential health impacts of pesticide residues. The deterministic method uses point estimates of residue levels, consumption rates, and toxicological reference values to calculate hazard quotients. In contrast, the probabilistic approach incorporates variability and uncertainty by using statistical distributions and Monte Carlo simulations, offering a more comprehensive evaluation of exposure scenarios and population risk [25,26,27].

This study introduces a novel, integrative framework for pesticide residue assessment that combines deterministic and probabilistic models, supported by sensitivity analysis and compound-specific risk ranking. Although numerous studies have addressed pesticide residues and related health risks in various vegetable types, investigations that simultaneously apply both deterministic and probabilistic approaches remain relatively scarce. Such methodologies have occasionally been applied to citrus fruits [27], apples [28], cucumbers, cantaloupes, and melons [29]; however, their application to root and tuber crops is notably limited [30]. Comprehensive risk assessments involving multiple modeling approaches are rarely conducted for these crop types. Roots, tubers, and bulb vegetables, due to their growth below ground, have unique exposure pathways and residue retention characteristics that are under-represented in risk assessments. This study addresses this gap by providing a comprehensive risk assessment for various types of root and tuber vegetables, as well as onions.

The novelty of this research lies in the simultaneous evaluation of both acute and chronic health risks for adults and children. Additionally, the quantitative pesticide risk ranking matrix, tailored to residue occurrence, toxicity, and exposure parameters, offers a refined prioritization tool that may inform national and international food safety policies. To the best of our knowledge, this is among the first studies to jointly apply these advanced methodologies to a broad spectrum of underground vegetables within a single framework, aiming to enhance consumer protection strategies in both local and broader regulatory contexts.

The specific aims of this study were to investigate the occurrence of pesticide residues in commonly consumed root vegetables and onions and to assess the potential acute and chronic health risks arising from their dietary intake. Exposure scenarios for both adults and children were quantified using deterministic calculations and Monte Carlo simulations, offering a more refined estimate of the consumer risk. The risk ranking of individual pesticides further supports targeted risk mitigation strategies and informed regulatory decisions.

## 2. Materials and Methods

### 2.1. Samples and Reagents

In this study, 580 vegetable samples were collected from the local markets in Serbia. The samples included vegetables like celery (*n* = 34), ginger (*n* = 60), potatoes (*n* = 273), radishes (*n* = 36), horseradish (*n* = 7), carrots (*n* = 20), onions (*n* = 44), and leeks (*n* = 106). All the samples were stored under refrigerated conditions at 4 °C until analysis. Market availability and distribution of vegetables during the sampling period were the main factors contributing to the unequal distribution of sample sizes among the vegetables. A larger number of potato samples were intentionally collected, as potatoes are the most widely consumed root vegetable, making them a key focus for assessing potential dietary exposure to pesticide residues. On average, individual vegetable units weighed between 8 and 900 g, depending on the type. For example, potato samples weighed 100–200 g, carrots 70–150 g, celery 500–900 g, onions 90–150 g, leeks 150–280 g, ginger 50–100 g, radishes 8–15 g, and horseradish 300–400 g. Each sample was analyzed as a whole or homogenized composite, depending on size and analytical requirements.

Certified reference standards were obtained from Restek (Bellefonte, PA, USA), Dr. Ehrenstorfer (Augsburg, Germany), and Lab Instruments (Castellana Grotte, Italy) (Table S1). HPLC-grade acetonitrile was purchased from Lachner (Neratovice, Czech Republic), whereas methanol, formic acid, and ammonium formate (with purity exceeding 99%) were obtained from Carlo Erba (Milan, Italy). Other reagents, including sodium chloride, anhydrous magnesium sulfate, disodium hydrogen citrate sesquihydrate (C_6_H_8_Na_2_O_8_), trisodium citrate dihydrate (C_6_H_5_O_7_Na_3_·2H_2_O), primary and secondary amine sorbents (PSAs), C18 material, and graphitized carbon black (GCB), were procured from Sigma–Aldrich (St Louis, MO, USA).

### 2.2. Pesticide Extraction and Analysis

Vegetable samples were homogenized using a high-capacity homogenizer, and 10 g of the homogenate was transferred into a 50 mL centrifuge tube. Pesticide residues were then extracted, and the extract was cleaned in accordance with the EN 15,662 method [31].

To ensure precise quantification and account for matrix-specific variations, pesticide residues were analyzed separately for each type of vegetable. Prior to the analysis, all vegetable samples were rinsed with tap water; peeling was not performed, except for onions, where the outer dry skin was removed. Approximately 1 kg of each vegetable sample was homogenized (Grindomix GM 300, Retsch, Haan, Germany—4.5 L, 1.1 kW) to ensure sample representativity, following the European Commission guidance on analytical quality control for pesticide residue analysis (SANTE/11312/2021) [32]. Briefly, 10 mL of acetonitrile and a mixture of buffering salts, comprising 0.5 g of disodium hydrogen citrate, 1 g of sodium chloride, 1 g of trisodium citrate dihydrate, and 4 g of magnesium sulfate, were added. The mixture was vigorously vortexed for 1.5 min and centrifuged at 3500 rpm for 3 min. The resulting supernatant was transferred to a clean tube for clean-up. For all analyzed samples (potato, onion, celery, carrot, ginger, radish, and horseradish), the clean-up sorbents included 0.90 g of magnesium sulfate and 0.15 g of PSA. Additionally, for carrot, ginger, radish, and horseradish, the clean-up mixture also contained 0.15 g of C18 and 0.054 g of GCB to effectively remove pigments, lipophilic compounds, and essential oils. After a second vortexing and centrifugation, the final extract was filtered using a 0.22 µm PTFE syringe filter. The prepared extracts were analyzed using HPLC-MS/MS and GC-MS/MS [33]. Each sample was processed and analyzed in triplicate.

Pesticide analysis using HPLC-MS/MS was performed to identify and quantify compounds with medium to high polarity and thermal instability (pyrimethanil, boscalid, tebuconazole, azoxystrobin, fluopyram, difenoconazole, tebufenpyrad, tebufenozide, iprovalicarb, clothianidin, fluopicolide, fosthiazate, fluazifop, epoxiconazole, dimethomorph, thiamethoxam, imidacloprid, cyromazine, fenhexamid, imazalil, metamitron, propamocarb, carbaryl, and isoprocarb). A Thermo Scientific Accela HPLC system coupled with a TSQ Quantum Access MAX triple quadrupole mass spectrometer (San Jose, CA, USA) was utilized, operating in multiple reaction monitoring (MRM) mode to ensure high sensitivity and selectivity. Chromatographic separation was achieved on an Accucore aQ C18 column (100 mm × 2.1 mm, 2.6 µm particle size, Thermo Scientific, Waltham, MA, USA) using gradient elution with water and methanol, both containing 0.1% formic acid and 5 mM ammonium formate as modifiers. The system was equipped with a heated electrospray ionization (HESI) interface, which operated in both positive and negative ionization modes depending on the physicochemical characteristics of the analytes. A gradient program was applied, starting with 100% A, transitioning to 70% B over 7 min, reaching 100% B at 9 min, and holding for 3 min, before returning to 100% A at 12.5 min and maintaining it for an additional 4 min. The column was maintained at 40 °C, with a flow rate of 0.3 mL/min and an injection volume of 10 µL. This method provided low detection limits and reliable quantification across a wide range of pesticide residues in complex food matrices such as root, tuber, and bulb samples.

For the determination of volatile and thermally stable pesticide residues (metolachlor, prosulfocarb, linuron, pirimiphos-methyl, chlorpropham, ethoxyquin, chlorpyrifos, resmethrin, and piperonyl butoxide), GC-MS/MS analysis was carried out using a Thermo Trace 1310 GC system linked to a TSQ 8000 Evo mass spectrometer and equipped with a TriPlus AS autosampler. The instrument was operated in electron ionization (EI) mode with detection in multiple reaction monitoring (MRM) to enhance specificity and minimize matrix interferences. Analytes were separated on a Trace TR Pesticide II capillary column (30 m × 0.25 mm ID × 0.25 µm film thickness, Thermo Scientific) optimized for pesticide determination. The injector operated in splitless mode to achieve maximum sensitivity, and a multi-step oven temperature program was applied to ensure optimal elution of compounds with varying volatilities. The injector temperature was programmed to increase from 75 °C (1 min hold) to 330 °C over 2 min. The oven temperature was initially held at 60 °C for 2.3 min, ramped to 90 °C at 25 °C/min with a 1.5 min hold, further increased to 180 °C at the same rate, then gradually increased to 280 °C at 5 °C/min, and finally increased to 300 °C at 10 °C/min with a 5 min hold. Helium (purity ≥ 99.999%) was used as the carrier gas at a constant flow rate of 1.20 mL/min. The temperatures of the transfer line and the ion source were maintained at 250 °C and 300 °C, respectively. The injection volume was 1 µL. This technique enabled the accurate identification and quantification of non-polar pesticides, effectively complementing the HPLC-MS/MS method for comprehensive multi-residue analysis.

### 2.3. Quality Assurance/Quality Control

The analytical method for pesticide residue determination in vegetable samples was validated following the SANTE/11312/2021 guidelines to ensure reliability and performance. The key validation parameters included linearity, matrix effects, selectivity, sensitivity, accuracy, and precision [32]. Calibration curves were constructed using pesticide standards dissolved in both pure solvent and matrix-matched extracts, covering a concentration range of 5–200 µg/L for both HPLC-MS/MS and GC-MS/MS analyses. A correlation coefficient (r^2^) greater than 0.99 was considered indicative of acceptable linearity. Blank matrices were confirmed by screening the samples in the absence of target pesticides before spiking. Matrix effects (MEs) were calculated as the ratio of the analytical signal obtained in the matrix to that in the solvent and expressed as a percentage to assess signal enhancement or suppression. Selectivity was confirmed by comparing the chromatograms of the blank and control samples to those containing spiked pesticides, ensuring no interference at the target retention times. Accuracy and precision were evaluated through recovery experiments by fortifying blank samples at two concentration levels (10 and 100 µg/kg), with six replicates (*n* = 6) at each level. Recovery rates were required to fall within the range of 70–120%, and precision was expressed as the relative standard deviation (RSD), which had to remain below 20%. The sensitivity of the method was determined by the limits of detection (LOD) and quantification (LOQ). The LOD and LOQ were calculated using an S/N of 3 and S/N of 10 in the lowest spiked recovery experiment.

### 2.4. Health Risk Assessment

#### 2.4.1. Prioritization of Pesticides Using Risk Ranking

To estimate the potential health risks posed by each detected pesticide, a risk ranking matrix developed by the UK Veterinary Residues Committee was employed [34]. The total residual risk score (S) was calculated using Equation (1).

(1) $$S=\left(A+B\right)\times \left(C+D+E\right)\times F$$

In this model, parameters A and B reflect toxicity, while C, D, E, and F capture various dimensions of human exposure. Specifically, A represents the acute toxicity score based on the oral LD_50_ values, classified into toxicity categories and sourced from the World Health Organization [35], US Environmental Protection Agency [36,37,38], or scientific opinion on hexachlorocyclohexanes [39]. B denotes chronic toxic potency, determined from the Acceptable Daily Intake (ADI) values available in the EU Pesticide Database [40], Pesticide Properties DataBase (PPDB) [41], or Joint FAO/WHO Meeting on Pesticide Residues (JMPR) database [42]. Parameter C quantifies dietary exposure by assessing the proportion of specific food items (e.g., root, tuber vegetable, or onion) in the total diet, using national consumption data from the Serbian Household Budget Survey [43]. D reflects the pesticide application frequency, calculated using the formula FOD = (N/T) × 100, where N is the number of pesticide treatments and T is the crop growth period in days. E captures the potential exposure of sensitive populations and was conservatively assigned a constant score (E = 3) due to limited demographic-specific exposure data. The final parameter, F, addresses the actual pesticide residue levels in relation to the established Maximum Residue Limits (MRLs) [40], using a weighted scoring system:

(2) $$F=\frac{F_{0}\times 1+F_{1}\times 2+F_{2}\times 3+F_{3}\times 4}{n}$$

Here, F_0_ represents the number of samples with no detectable residues, F_1_ those with residues < 1 × MRL, F_2_ for residues between 1 and 10 × MRL, and F_3_ those exceeding 10 × MRL. The average F score was used to account for contamination severity across all analyzed samples.

Each pesticide was evaluated and ranked based on its total score, with higher scores indicating greater potential risk. Complete definitions, scoring criteria, and supporting values (e.g., LD_50_, ADI, and MRL) are provided in Supplementary Tables S2–S6.

#### 2.4.2. Chronic and Acute Risk Assessment

Acute dietary risk (short-term risk) was estimated using the International Estimated Short-Term Intake (IESTI) model [44], which calculates the intake of pesticide residues over a single day and compares it with the established Acute Reference Dose (ARfD, mg/kg body weight) [40,45,46,47]. The percentage of the ARfD (%ARfD) was determined using parameters such as the highest residue concentration (HR), large portion consumption (LP; 97.5th percentile of eaters), unit edible portion (Ue), and a variability factor (v = 3), adjusted for different age groups (bw: body weight). Two formulas were used depending on whether the unit portion was higher or lower than LP. For adult consumers, the acute dietary risk assessment was conducted using case 2a (Equation (3)) of the IESTI model for ginger, potato, leek, and carrot, while case 2b (Equation (4)) was applied for celery, onion, horseradish, and radish. In the case of children, case 2b was consistently used for all assessed vegetable types. If the %ARfD exceeded 100% (or hazard quotient acute, HQa, exceeded 1), short-term exposure was considered unacceptable.

Case 2a: 25 g ≤ Ue < LP

(3) $$\mathrm{IESTI}=\frac{\left(\left(U_{e}\times \mathrm{HR}\times v\right)+\left(\mathrm{LP}-U_{e}\right)\times \mathrm{HR}\right)}{\mathrm{bw}}$$

Case 2b: 25 g ≤ Ue > LP

(4) $$\mathrm{IESTI}=\frac{\mathrm{LP}\times \mathrm{HR}\times v}{\mathrm{bw}}$$

(5) $$\mathrm{HQa}=\frac{\mathrm{IESTI}}{\mathrm{ARfD}}$$

(6) %ARfD=HQa×100

Chronic risk was evaluated by estimating the Estimated Daily Intake (EDI, mg/kg bw) of each pesticide, based on the supervised trials median residue (STMR) and average food daily intake (Fi, kg/day), normalized by body weight (bw). The average daily intake was determined using national consumption data from the Serbian Household Budget Survey [44]; a portion for children was calculated as 40% of the adult portion [48]. Chronic risk was then expressed as a percentage of the Acceptable Daily Intake (%ADI), calculated by comparing the EDI to the respective ADI values (mg/kg bw). A %ADI below 100% (or hazard quotient chronic, HQc < 1) indicated that long-term exposure did not pose a health concern. Both the %ARfD and %ADI were used to assess overall dietary risk. Values exceeding 100% suggested an unacceptable risk, while lower values indicated an acceptable safety margin for consumers. These calculations provided a comprehensive evaluation of dietary risks, accounting for both high-consumption scenarios and long-term exposure patterns across different population groups.

(7) $$\mathrm{EDI}=\frac{\mathrm{STMR}\times \mathrm{Fi}}{\mathrm{bw}}$$

(8) $$\mathrm{HQc}=\frac{\mathrm{EDI}}{\mathrm{ADI}}$$

(9) %ADI=HQc×100

The cumulative chronic hazard index (HIc) and cumulative acute hazard index (HIa) were determined using Equation (10):

(10) HI=∑HQ

These cumulative risk values represent the total sum of the chronic (HQc) or acute (HQa) hazard quotients for all pesticides detected within a single vegetable sample. If the HI exceeds 1 (meaning that the %HI is greater than 100%), it indicates an increased likelihood of potential adverse health effects from pesticide exposure. In such circumstances, food products may be considered unsuitable for consumption.

### 2.5. Monte Carlo Risk Simulation

Uncertainty and sensitivity analyses related to chronic dietary risk assessments were conducted using a Monte Carlo simulation approach [25]. The simulations were performed using Oracle Crystal Ball software (version 11.1.3.0.0, Oracle Inc., Redwood Shores, CA, USA), employing 10,000 iterations to ensure the robustness and reliability of the results. In the simulation process, different probability distributions were assigned to the input variables: residue concentrations and body weight were modeled using a lognormal distribution to account for their right-skewed nature, while ingestion rates were modeled using a triangular distribution. The input variables without assigned probability distributions were treated as fixed-point values throughout the Monte Carlo simulations. This probabilistic method allows for a comprehensive evaluation of the variability and uncertainty inherent in chronic risk assessment.

## 3. Results and Discussion

### 3.1. Method Validation

Excellent linearity was observed for all the analyzed pesticides, with correlation coefficients (R^2^) ranging from 0.9935 to 0.9992 (Table 1). Limits of detection (LODs) were between 0.8 and 3.4 µg/kg, while limits of quantification (LOQs) did not exceed 10 µg/kg. Accuracy and precision were confirmed by recovery experiments conducted at two spiking levels. The recovery rates ranged from 84.7% (boscalid) to 115% (tebuconazole) at lower fortifications, with relative standard deviations (RSDs) between 1.8% (azoxystrobin) and 14.3% (tebufenpyrad). At higher spiked concentrations, the recoveries varied between 92.5% (pyrimethanil) and 114% (chlorpropham), while the RSD values ranged from 3.2% (azoxystrobin) to 11.9% (cyromazine). These validation results satisfied the acceptance criteria for pesticide residue analysis (mean recovery within 70–120% and RSD below 20%). Based on the calculated results, the investigated pesticides exhibited predominantly low to moderate matrix effects, ranging from −44.1 to +55.8 %. Most compounds displayed either medium or low matrix effects. Thus, matrix-matched calibration was employed to enhance the quantification accuracy.

### 3.2. Pesticide Distribution

**Figure 1:**
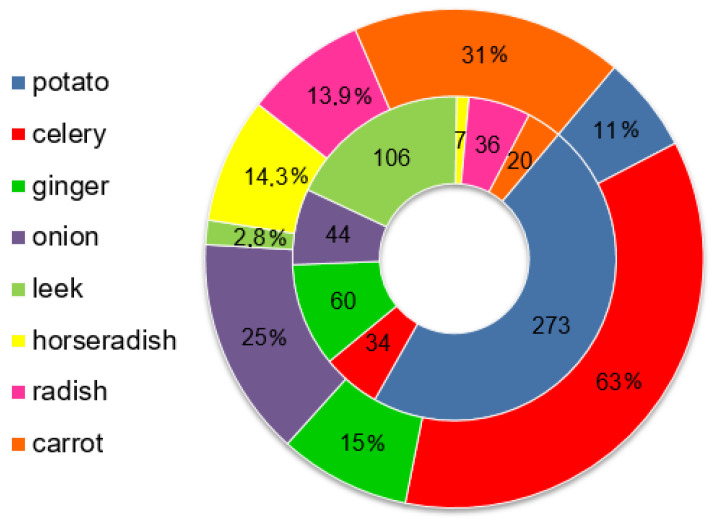
Number of analyzed root, tuber, and onion vegetable samples (inner circle) and percentage of positive samples per vegetable group (outer circle).

A total of 580 vegetable samples were examined for pesticide residues, confirming the presence of 33 different active substances in the 86 samples. Positive samples contained at least one residue, with concentrations ranging from 0.01 mg/kg (boscalid) to 9.8 mg/kg (metolachlor). The detected pesticides included 13 fungicides (pyrimethanil, boscalid, tebuconazole, azoxystrobin, fluopyram, difenoconazole, iprovalicarb, fluopicolide, epoxiconazole, dimethomorph, fenhexamid, imazalil, and propamocarb), 12 insecticides (carbaryl, resmethrin, tebufenpyrad, tebufenozide, pirimiphos-methyl, clothianidin, fosthiazate, thiamethoxam, imidacloprid, cyromazine, chlorpyrifos, and isoprocarb), 6 herbicides (metolachlor, prosulfocarb, linuron, chlorpropham, fluazifop, and metamitron), 1 synergist (piperonyl butoxide), and 1 non-classical pesticide (ethoxyquin). Piperonyl butoxide is primarily used as a synergist to boost the effectiveness of insecticides, especially pyrethrins and pyrethroids [49]. Ethoxyquin is mainly applied as an antioxidant preservative in animal feed and, to a lesser extent, as a post-harvest treatment to reduce spoilage in certain fruits [50]. Figure 1 illustrates the number of analyzed samples across different vegetable groups (inner circle) and the percentages of positive samples within each group of vegetables (outer circle). The outer circle shows the extent of pesticide contamination in each vegetable group. Potatoes exhibited the highest number of pesticides (16 compounds), followed by onions (11 compounds), celery (11 compounds), and radishes (6 compounds). Fewer residues were detected in ginger, carrot (five compounds), leek (four compounds), and horseradish (one compound). Details regarding the specific pesticides detected in each vegetable type are provided in Table 2.

In terms of contamination, celery exhibited the highest percentage of contaminated samples (63%), as well as the greatest variety of pesticides relative to the number of analyzed samples. Carrots and bulb onions followed, with 31% and 25% of the samples testing positive, respectively. For the remaining vegetables, the proportion of positive samples ranged from 2.8 to 15%. In comparison with previous investigations, our study revealed a slightly higher proportion of pesticide-positive celery samples than that reported by Fang et al. (2015) [51], who detected residues in 58% of celery samples collected in China. In contrast, a much lower level of contamination was observed for carrots in the study by Kazimierczak et al. (2022) [52] conducted in Poland, where only 5.0% of the samples tested positive. Cui et al. (2024) [21] reported a considerably higher contamination rate for ginger in China, with 66.5% of the samples containing pesticide residues. Regarding onion, the proportion of positive samples in our study was comparable to findings from Ethiopia reported by Jirata et al. (2024) [53], where 22–28% were contaminated. Chu et al. (2023) observed a slightly higher contamination rate in China [54]. Potato, the most consumed vegetable among those analyzed, showed a lower pesticide residue rate in our study than the 26.2% positive samples reported in the 2020 EU report [55]. Root and tuber vegetables such as carrots and potatoes are generally regarded as less problematic in terms of pesticide residues compared to leafy and fruiting vegetables. Potatoes are root vegetables regularly monitored by EFSA because of their high consumption levels [56], as well as their specific characteristics that involve pesticide applications at various stages of production. Pesticide residues may remain on the peel of the tubers and sometimes in their inner tissues due to their physiological characteristics, cultivation practices, post-harvest treatments with fungicides and sprout inhibitors, as well as due to storage in facilities with a history of pesticide usage [56,57,58]. However, according to recent EFSA reports, only 0.8% of potato samples in the EU exceeded the maximum residue level (MRL) in 2020, meaning that the majority of samples adhere to regulatory limits. Carrots and onions are also among the commodities routinely monitored under the EU-coordinated multiannual control program (EU MACP) [59]. However, monitoring data suggest that these vegetables generally present a low pesticide-related risk, with MRL exceedances observed in only 1.2% of the carrot samples and 0.2% of the onion samples in 2020. Therefore, they pose minimal risks in terms of pesticide residue exposure [59].

Among the positive samples, nineteen contained multiple pesticide residues: one sample contained five pesticides (bulb onion), one contained four (celery), four contained three, and thirteen contained two. This frequent co-occurrence highlights the widespread use of pesticide mixtures and the need for a cumulative dietary risk assessment. The most frequently detected pesticide was azoxystrobin (14 samples), followed by boscalid (10 samples). Tebufenpyrad had the highest number of MRL (Supplementary Materials, Table S4) exceedances among all detected pesticides, exceeding the legal limit in seven ginger samples (MRL = 0.01 mg/kg). MRL exceedances were recorded for 29 samples, including celery (*n* = 5), ginger (*n* = 8), potato (*n* = 9), bulb onion (*n* = 3), leek (*n* = 1), horseradish (*n* = 1), radish (*n* = 1), and carrot (*n* = 1). In previous studies, Danek et al. (2021) [6] observed multi-residue presence in 8 of 15 potato samples, while Cui et al. (2024) [21] reported that 38.8% of ginger samples contained multiple residues. Elevated pesticide concentrations above the MRLs have also been reported in other studies, such as P et al. (2022) [24] and Kazimierczak et al. (2022) [52], the latter noting chlorpyrifos exceedance in organically grown carrots.

**Figure 2:**
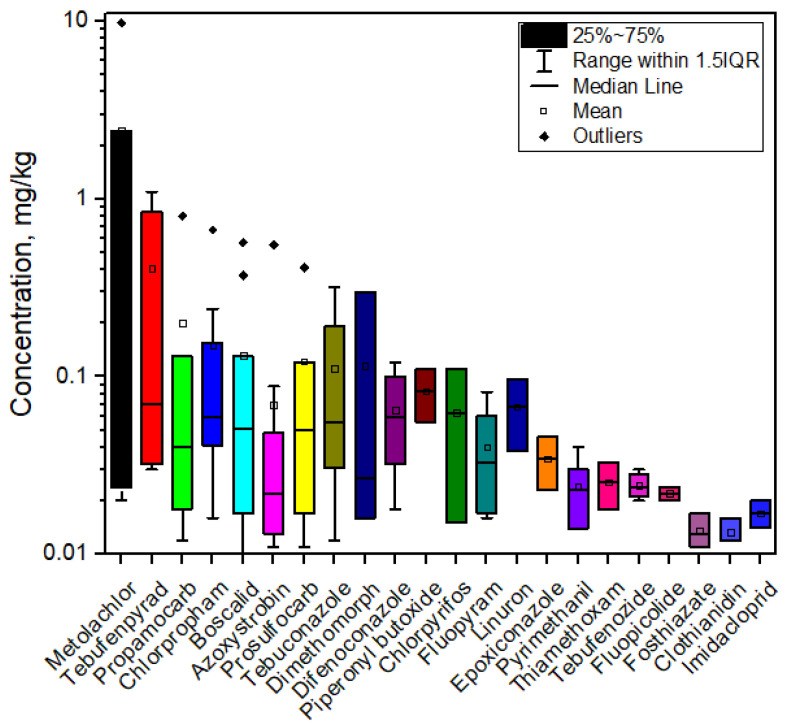
Box plots showing pesticide concentration distributions in root vegetables and onions (logarithmic scale).

Summary statistics (Supplementary Table S7) revealed that mean pesticide concentrations ranged from 0.013 mg/kg (clothianidin) to 2.45 mg/kg (metolachlor). The distribution profiles of the individual pesticides are shown in Figure 2 (box plots, logarithmic scale), illustrating substantial variability. To improve clarity and focus on the more frequently occurring residues, pesticides detected in only a single sample, specifically imazalil, fluazifop, isoprocarb, carbaryl, pirimiphos-methyl, metamitron, ethoxyquin, resmethrin, iprovalicarb, cyromazine, and fenhexamid, were excluded from the plot. Metolachlor exhibited the highest mean (2.45 mg/kg) and maximum (9.8 mg/kg) concentrations, followed by notable residues of tebufenpyrad (1.10 mg/kg), imazalil (0.97 mg/kg), propamocarb (0.80 mg/kg), chlorpropham (0.67 mg/kg), boscalid (0.57 mg/kg), and azoxystrobin (0.55 mg/kg). Moderate maximum concentrations were observed for prosulfocarb (0.41 mg/kg), tebuconazole (0.32 mg/kg), dimethomorph (0.30 mg/kg), and fluazifop (0.13 mg/kg). These results emphasize the necessity for regular monitoring programs and cumulative risk assessment strategies for pesticide residues in vegetables.

### 3.3. Cluster Analysis of Pesticide Residues

**Figure 3:**
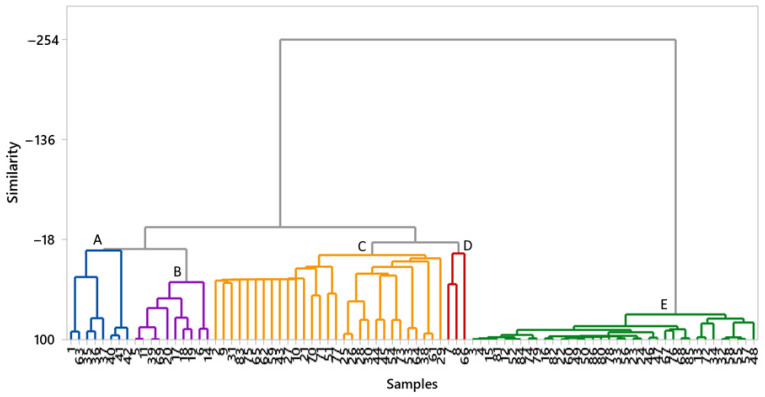
Cluster analysis of underground vegetables based on pesticide residues.

Hierarchical cluster analysis (HCA) was performed to explore the similarity in pesticide residue profiles among vegetable samples. The analysis was performed using Ward’s linkage method and Pearson’s distance as dissimilarity measures [60]. The resulting dendrogram (Figure 3) grouped the samples into five clusters (A–E), based on compositional similarities in the detected pesticide concentrations (Supplementary Materials, Table S8). Cluster A predominantly consisted of potato samples, indicating a high degree of internal homogeneity, likely attributable to uniform agricultural practices, similar cultivars, or comparable environmental growth conditions [61]. Cluster B was mainly composed of celery samples, which also displayed consistent characteristics within this group. In contrast, Cluster C exhibited a more heterogeneous composition, comprising a mixture of potatoes, ginger, onions, celery, isolated carrot samples, and horseradish. This diversity suggests potential overlap in chemical properties or pesticide residue patterns across these vegetables, possibly resulting from shared environmental exposures or cross-contamination during cultivation or processing [58]. Cluster D was relatively small, consisting of two celery samples and one onion, indicating localized similarity among these samples. Cluster E was the most compositionally diverse, encompassing various vegetable types, including potatoes, celery, onions, ginger, carrots, and radishes. Several sub-clusters were observed within this cluster, with the leftmost sub-cluster primarily containing potato samples. The grouping observed in this cluster may reflect broader or overlapping residue profiles, potentially due to mixed production sources or greater variability in agricultural inputs. These mixed clusters indicate that certain pesticide profiles are common across multiple vegetable types, likely reflecting the use of similar pesticide treatments or environmental factors that affect diverse crops within the same agricultural system.

Overall, cluster analysis revealed crop-specific residue patterns for potato and celery, whereas mixed clusters indicated potential cross-contamination or shared sources of pesticide exposure. These findings emphasize the need for crop-specific monitoring strategies and underscore the relevance of cluster-based classification for assessing pesticide distribution in complex food matrices.

### 3.4. Risk Ranking

**Figure 4:**
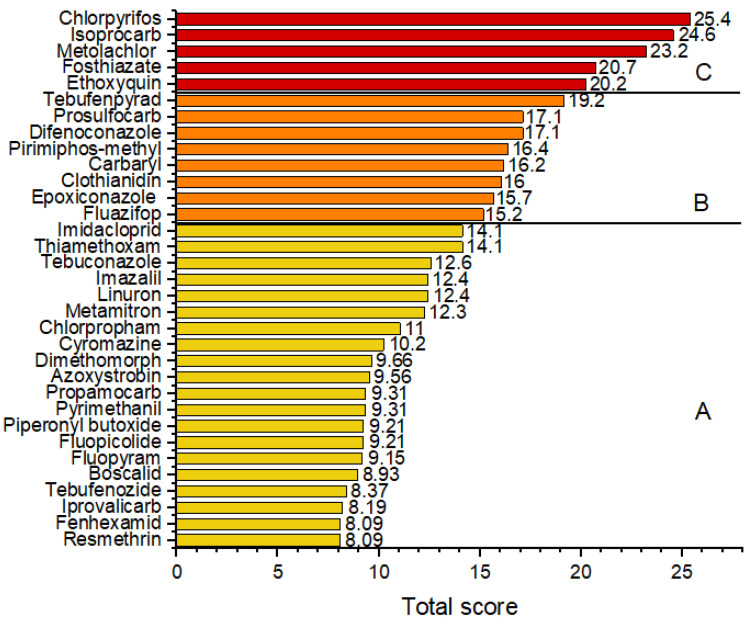
Risk ranking of pesticide residues detected in root vegetables and onions based on total risk scores of vegetables purchased in Serbia. A—low-risk group, B—medium-risk group, and C—high-risk group.

Figure 4 and Supplementary Table S5 present the risk ranking of the pesticide residues based on their total scores. The pesticides were categorized into three groups according to their risk levels. Group C, which included chlorpyrifos, isoprocarb, metolachlor, fosthiazate, and ethoxyquin, represents compounds with the highest total scores, indicating a high potential health risk. The pesticides in this group had overall risk scores above 20, accounting for 15.1% of the detected pesticides. These pesticides also exceeded MRL values or had low LD_50_ or ADI values. Group B consisted of pesticides with moderate risk scores, with the total risk score ranging from 15.0 to 19.9. This group included tebufenpyrad, prosulfocarb, difenoconazole, pirimiphos-methyl, carbaryl, clothianidin, epoxiconazole, and fluazifop (24.2% of the pesticides detected). Group A included the majority of the analyzed pesticides (60.6% of the detected pesticides), characterized by lower total scores and a relatively lower health risk. The low-risk group comprised pesticides with total scores below 15.0. This classification highlights the need for closer monitoring of Group C compounds because of their greater contribution to potential dietary exposure risks. The pesticides detected in this study included 15 unapproved substances. Among these, four were classified as high risk (chlorpyrifos, isoprocarb, metolachlor, and ethoxyquin), four as medium risk (carbaryl, clothianidin, epoxiconazole, and fluazifop), and seven as low risk (imidacloprid, thiamethoxam, linuron, chlorpropham, cyromazine, dimethomorph, and resmethrin).

**Figure 5:**
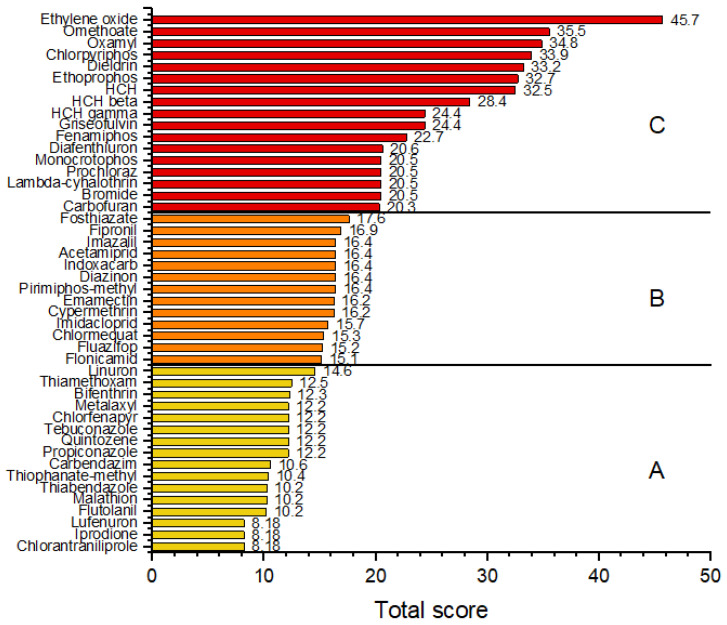
Risk ranking of pesticide residues in root vegetables and onions based on total risk scores. Data are derived from RASFF notifications (2020–2025). Pesticides were categorized into three risk groups: A—low-risk group, B—medium-risk group, and C—high-risk group.

Figure 5 and Supplementary Table S6 present the classification of pesticides detected in root vegetables and onions based on data from the RASFF database for the period 2020–2025 [62]. Since the samples included in this dataset mostly involved cases where MRLs were exceeded, a significantly higher number of pesticides were classified as high risk. Additionally, the database includes pesticides that are banned from use in the European Union. During the observed period, 181 samples were reported in the RASFF system, and 46 different pesticides were identified. Seventeen pesticides were classified as high risk (37% of reported pesticides), including ethylene oxide, omethoate, oxamyl, chlorpyrifos, dieldrin, ethoprophos, HCH, beta-HCH, gamma-HCH, griseofulvin, fenamiphos, diafenthiuron, monocrotophos, prochloraz, lambda-cyhalothrin, bromide, and carbofuran. Among these, eight pesticides—ethylene oxide, chlorpyrifos, dieldrin, HCH, beta-HCH, gamma-HCH (lindane), monocrotophos, and carbofuran—are explicitly banned in the EU [40]. Additionally, the remaining high-risk pesticides (with total risk scores ≥ 20) were not approved for use in the EU, except for lambda-cyhalothrin. The root vegetable samples listed in the RASFF database were primarily imported products from non-EU countries, predominantly from Asia and Africa. The medium-risk group included 13 pesticides, comprising banned/non-approved (diazinon, fipronil, indoxacarb, imidacloprid, and fluazifop) and approved substances (fosthiazate, imazalil, pirimiphos-methyl, acetamiprid, emamectin, cypermethrin, chlormequat, and flonicamid) for use in the European Union [40]. The low-risk pesticide group included both non-approved (linuron, thiamethoxam, bifenthrin, chlorfenapyr, quintozene, propiconazole, carbendazim, thiophanate-methyl, lufenuron, and iprodione) and approved pesticides (metalaxyl, tebuconazole, thiabendazole, malathion, flutolanil, and chlorantraniliprole).

### 3.5. Acute and Chronic Risks of Pesticide Residues in Root Vegetables and Onions

The average values for acute (short-term intake) risk (HQa) ranged from 1.13 × 10^−6^ for metamitron to 1.53 × 10^−2^ for tebufenpyrad. The corresponding chronic (long-term intake) risk values (HQc) ranged from 6.03 × 10^−7^ for fenhexamid to 2.02 × 10^−3^ for tebufenpyrad (Supplementary Materials, Table S9). These findings suggest that the detected pesticide residues are unlikely to pose either short- or long-term health risks through dietary exposure. However, this applies to the presence of individual pesticides, while a realistic risk assessment requires considering the combined presence of all pesticides in a single sample, that is, evaluating the total cumulative risk. The mean, maximum, and minimum values of total acute and chronic health risks for both adults and children are presented in Table 3 and Table 4, respectively. For adults, the acute hazard index (HIa) ranged from 1.66 × 10^−4^ to 5.11 × 10^−1^, corresponding to %ARfD values between 0.0166% and 51.1%. These results indicated that all analyzed samples were within the safe limits for consumption (HIa < 1 or %ARfD < 100%). However, the maximum acute risk values indicate potential concern associated with higher consumption of ginger. An acute risk assessment for adults revealed that three ginger samples, each containing pesticide residues exceeding their respective MRLs, had acute risk values of 51.1, 39.3, and 33.3%. For children, HIa values ranged from 6.23 × 10^−5^ to 6.31 × 10^−1^ and for %ARfD from 0.00623 to 63.1%. The highest acute risk in children (63.1%) was observed in a celery sample that also exceeded the MRL limits.

The observed differences in acute risk (Table 3) between adults and children are attributed to variations in dietary intake patterns, particularly the lower consumption of ginger among children than among adults. Regarding chronic exposure (Table 4), the chronic hazard index (HIc) for adults ranged from 1.80 × 10^−5^ to 5.90 × 10^−2^ (corresponding to %ADI values between 0.00180% and 5.90%) and for children from 3.36 × 10^−5^ to 1.10 × 10^−1^ (0.00336% to 11.0% of the ADI). These findings indicate that cumulative chronic dietary exposure to pesticide residues from the consumption of root vegetables does not pose a health risk, as the %ADI values remained well below 100% for both adults and children in Serbia, based on average intake levels. In the present study, although MRL exceedances were observed in 29 samples, both the acute and chronic health risks remained low in most cases. This can be attributed to the relatively low dietary intake of the most affected vegetables, namely celery and ginger.

Our findings are consistent with those of previous studies. For example, a health risk assessment of pesticide residues in celery conducted in China reported chronic and acute risks for adults within acceptable limits (%ADI ranged from 0.01 to 1.80% and %ARfD from 0.05 to 28%) [51]. Similar conclusions were drawn in studies from Greece [63] and Turkey [64,65], which also found no significant health risks associated with dietary exposure to pesticide residues in vegetables. For example, in a study from Greece, the acute risk (HQa) for celery and potatoes was 0.0130 and 0.0113, respectively, while the chronic risk (HQc) for the same types of vegetables was 0.117 and 0.0446. These values are well below the threshold of 1, indicating a low health risk [63]. On the other hand, a risk assessment study on pesticide residues in onions conducted in China [54] concluded that although the majority of samples met safety standards, acute health risks were identified for cyhalothrin (%ARfD was 297.95% in children and 140.17% in adults) and carbofuran (%ARfD was 107.94% for children). Similarly, a study on radishes reported that while the HQ and cumulative risk index were well below the threshold, the %ARfD for triazophos exceeded 100 for all age groups (%ARfDs for adults, children, and toddlers were 203.38, 241.0, and 533.97%, respectively), indicating a potential acute risk [22]. Additionally, the acute risk for toddlers was exceeded for carbofuran, aldicarb, monocrotophos, and parathion, with %ARfD values of 152.23, 179.16, 178.57, and 135.83, respectively.

Pesticide exposure from root and tuber vegetables can vary significantly depending on individual dietary habits, with frequent or high-volume consumption potentially leading to greater intake. However, several studies have demonstrated that common household processing methods—such as washing, peeling, and thermal treatment (e.g., boiling or frying)—can substantially reduce pesticide residue levels in these vegetables. For instance, Terfe et al. (2023) reported that peeling and boiling potatoes led to near-complete removal of organochlorine pesticides [66]. Phopin et al. (2022) showed that boiling, blanching, and stir-frying reduced pesticide residues by 18–100% in vegetables, with thermal treatments being especially effective [67]. Similarly, Chu et al. (2024) found that boiling is the most effective method for reducing boscalid levels in Welsh onions [68]. According to Jensen et al. (2022), even high vegetable consumers remain within safe exposure limits, particularly when the food is properly processed [69].

**Figure 6:**
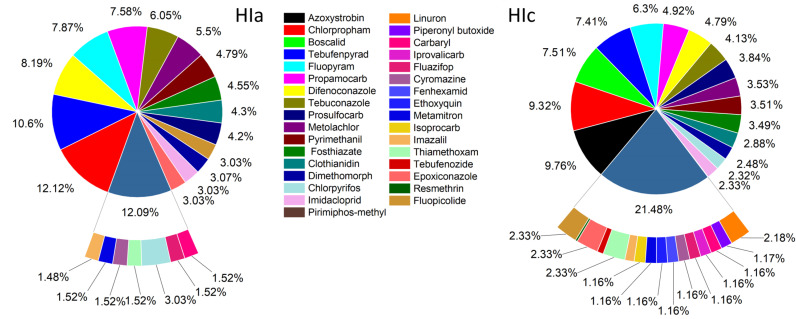
Contribution of pesticides to estimated acute and chronic risks.

Figure 6 illustrates the contribution of individual pesticides to the overall acute and chronic health risk. The most influential compounds for acute risk were chlorpropham, tebufenpyrad, difenoconazole, fluopyram, and propamocarb. In terms of chronic risk, the highest contributions were associated with azoxystrobin, chlorpropham, boscalid, tebufenpyrad, and fluopyram. An acute risk assessment was performed for 23 detected pesticides; the remaining compounds (*n* = 10) were excluded (boscalid, azoxystrobin, linuron, resmethrin, tebufenozide, iprovalicarb, piperonyl butoxide, fenhexamid, ethoxyquin, and isoprocarb) from the acute risk evaluation due to the absence of established ARfD values.

### 3.6. Monte Carlo Simulation

**Figure 7:**
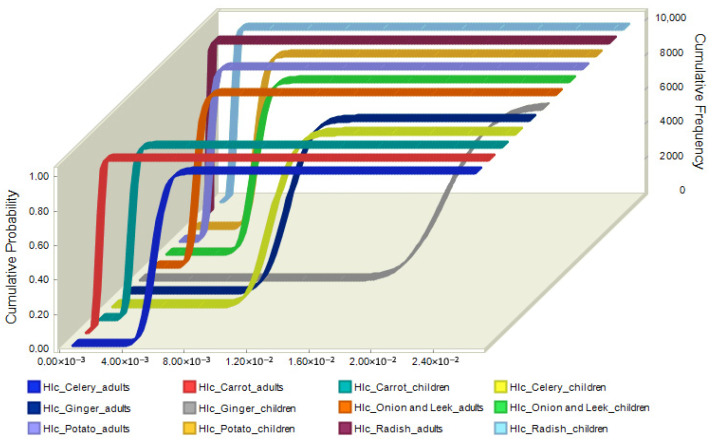
Monte Carlo simulation for chronic health risk for adults and children.

Figure 7 illustrates the cumulative probability distributions of the chronic hazard index (HIc) for various vegetables and population groups (adults and children), based on the Monte Carlo simulation results. Children exhibit higher HIc values than adults for all vegetables, indicating a greater risk of chronic dietary exposure [48]. Ginger and celery had the highest HIc values among adults and children. On the other hand, carrots and radishes generally had the lowest HIc values. For adults, the HIc ranged from approximately 6.48 × 10^−4^ (10th percentile) to 1.25 × 10^−2^ (90th percentile), indicating low variability and a low overall risk (Supplementary Table S10). In contrast, children exhibited higher risk values, with the HIc ranging from 1.21 × 10^−3^ to 2.33 × 10^−2^ (Table S10). Although all values remained well below the safety threshold (HIc < 1), the results confirmed that children are more vulnerable to chronic pesticide exposure, primarily due to their lower body weight and higher intake relative to body mass. This finding highlights the importance of including sensitive population groups in dietary risk assessments.

**Figure 8:**
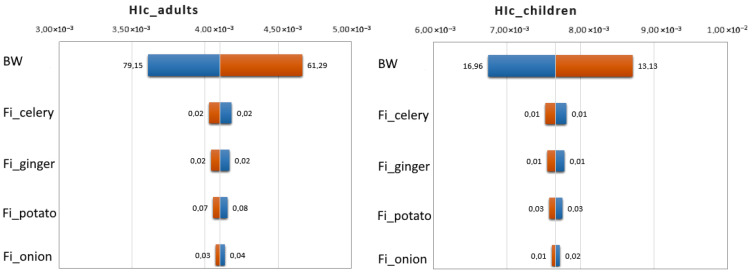
Sensitivity analysis by tornado chart for chronic health risk from pesticides in root vegetables.

Figure 8 presents the results of a sensitivity analysis for the total chronic health risk (HIc) associated with pesticide residues in root vegetables, conducted separately for adults (A) and children (B). The analysis identified the most influential input parameters that affect the variability in the risk estimates. For both population groups, body weight (BW) was identified as the most influential factor, followed by ingestion of celery, ginger, potato, and onion + leek. Body weight was the most sensitive parameter in the model, and even small variations could significantly affect the final HIc value. This is expected, as body weight is in the denominator of the risk equation (EDI = STMR × Fi/BW), meaning that a lower body weight results in a higher estimated risk. Based on the length of the left bar (orange) in the tornado chart for body weight, it can be observed that the HIc is slightly more sensitive to an increase in body weight than to a decrease. The higher influence of celery and ginger ingestion rates on chronic health risk was due to the higher contamination of these vegetables compared to other types of vegetables. The influence of these parameters is followed by the ingestion of potatoes, the root vegetables most consumed by the average population.

## 4. Conclusions

This study provided a comprehensive assessment of pesticide residues in commonly consumed root vegetables and onions by combining advanced analytical techniques with a probabilistic health risk assessment model. A total of 33 pesticide residues were identified across 580 vegetable samples, with multiple residues and MRL exceedances observed in 19 and 29 samples, respectively. Although individual pesticides generally did not pose significant health risks, cumulative risk analysis revealed that certain samples, particularly ginger and celery, may contribute more substantially to dietary exposure. Monte Carlo simulations confirmed that chronic exposure levels for both adults and children remained below acceptable thresholds, with children identified as the more vulnerable group. Sensitivity analysis further emphasized the critical influence of body weight and vegetable consumption rates on risk estimates. The findings underscore the importance of continuous monitoring, stricter enforcement of pesticide regulations, and implementation of cumulative exposure assessments in routine food safety evaluations. Although dietary patterns were not evaluated in this study, the results may bolster public health initiatives that focus on mitigating dietary risks. Strategies such as promoting dietary diversification, educating consumers on portion sizes and consumption frequency, and addressing the requirements of vulnerable populations, particularly children, could be considered.

## Figures and Tables

**Table 1:** Validation of GC-MS/MS and LC/MS/MS methods for analysis of pesticide residues in vegetable samples.

Pesticide	ME %	R^2^	LOD µg/kg	R ^#^ %	RSD ^#^ %	R ^##^ %	RSD ^##^ %
Celery							
Pyrimethanil	−27.4	0.9936	1.2	106	2.64	92.5	9.31
Boscalid	9.30	0.9955	0.9	94.7	5.75	110	3.95
Tebuconazole	15.5	0.9962	1.5	115	9.51	93.3	5.78
Metolachlor	−10.3	0.9992	2.7	111	7.34	105	11.1
Azoxystrobin	−13.2	0.9940	1.4	101	1.80	97.8	8.46
Fluopyram	11.3	0.9988	1.2	87.7	2.45	93.1	9.74
Difenoconazole	16.8	0.9939	2.9	89.5	11.2	113	10.1
Prosulfocarb	−33.5	0.9943	1.8	99.7	3.12	105	11.5
Linuron	−10.5	0.9937	1.5	104.3	7.78	95.1	7.35
Carbaryl	12.4	0.9953	1.4	114	8.20	94.2	3.65
Resmethrin	−28.4	0.9982	1.9	93.2	11.3	102	7.12
Potato							
Pyrimethanil	11.5	0.9991	1.6	102	2.43	94.3	4.95
Metolachlor	−27.1	0.9963	2.5	109	4.01	103	6.01
Azoxystrobin	27.3	0.9987	1.3	98.3	3.98	95.4	4.75
Fluopyram	−15.2	0.9974	2.3	90.1	3.40	97.2	5.20
Chlorpropham	35.0	0.9991	2.5	88.0	3.33	114	6.62
Clothianidin	45.2	0.9989	1.0	94.1	12.4	93.9	10.0
Fluopicolide	−44.1	0.9948	3.1	113	4.98	113	3.94
Fosthiazate	17.6	0.9935	2.2	95.2	10.2	101	5.51
Fluazifop	38.3	0.9953	1.4	97.9	13.7	97.2	3.20
Epoxiconazole	16.2	0.9972	1.2	103	6.31	92.9	7.70
Dimethomorph	31.4	0.9990	2.8	111	4.05	101	3.90
Thiamethoxam	32.2	0.9937	3.0	85.9	6.72	93.3	5.19
Piperonyl butoxide	48.3	9.9986	1.4	90.2	2.90	94.0	4.20
Imidacloprid	55.8	0.9935	0.8	91.8	11.0	110	7.32
Cyromazine	−17.3	0.9974	1.9	86.5	7.83	105	11.9
Horseradish							
Metamitron	−18.4	0.9990	1.1	89.8	3.38	92.8	8.15
Ginger							
Metolachlor	−24.5	0.9981	2.7	112	5.15	110	12.4
Tebufenpyrad	−55.6	0.9935	1.1	95.1	14.3	107	5.52
Tebufenozide	−23.1	0.9985	1.5	105	3.50	111	7.17
Iprovalicarb	−29.9	0.9949	2.7	110	7.91	97.3	3.85
Pirimiphos-methyl	22.8	0.9951	1.3	93.7	10.5	93.0	5.19
Onion							
Fluopicolide	−7.5	0.9958	2.6	91.2	5.91	104	4.91
Dimethomorph	−8.8	0.9977	1.3	85.3	3.89	94.9	6.30
Piperonyl butoxide	13.5	0.9964	3.1	99.1	1.99	98.3	3.73
Imidacloprid	−13.3	0.9986	0.9	103	6.01	105	3.79
Cyromazine	−15.6	0.9974	3.2	94.7	7.20	104	11.9
Fenhexamid	−11.2	0.9943	2.3	112	3.34	111	9.91
Ethoxyquin	12.2	0.9983	0.9	87.4	5.07	103	5.54
Imazalil	25.1	0.9949	3.4	114	9.63	93.5	4.11
Chlorpyrifos	21.1	0.9986	2.5	95.7	13.4	113	11.0
Propamocarb	−7.0	0.9940	3.0	92.1	2.95	98.1	9.78
Radish							
Boscalid	−12.2	0.9973	1.2	84.7	3.96	97.2	12.8
Azoxystrobin	−14.5	0.9951	1.7	98.3	7.05	110	3.20
Thiamethoxam	−11.1	0.9959	2.9	104	8.70	112	6.86
Chlorpyrifos	14.8	0.9968	2.3	93.5	4.12	94.2	7.33
Propamocarb	−8.7	0.9973	1.6	107	5.95	112	11.5
Carrot							
Boscalid	−15.9	0.9984	1.1	93.9	8.14	98.0	5.60
Tebuconazole	−33.5	0.9975	1.7	95.1	4.83	107	4.77
Azoxystrobin	−6.4	0.9961	1.2	105	6.24	113	5.95
Prosulfocarb	18.1	0.9992	2.0	111	5.13	105	8.48
Isoprocarb	11.0	0.9957	2.8	89.0	11.6	94.9	2.85

^#^—Spiking level 10 µg/kg; ^##^—spiking level 100 µg/kg; ME—matrix effect; LOD—limit of detection; RSD—relative standard deviation; R—recovery; R^2^—coefficient of determination.

**Table 2:** Pesticides detected in the analyzed vegetables.

Potatoes	Onion	Celery	Radish	Ginger	Carrot	Leek	Horseradish
PYM	PYM	PYM	BOS	MTL	BOS	BOS	MTT
MTL	BOS	BOS	AZX	TBP	TEB	TEB	
AZX	AZX	TEB	TMX	TBZ	AZX	AZX	
FPM	FPM	MTL	CPF	IPV	PSC	PBO	
CHP	FPC	AZX	MTT	PPM	ISP		
CLT	DMM	FPM	PPC				
FPC	IMI	DFZ					
FST	FHX	PSC					
FLP	ETQ	LIN					
EPX	IMZ	CRB					
DMM	CPF	RMT					
TMX							
PBO							
IMI							
CYR							
PPC							

Metolachlor (MTL), tebufenpyrad (TBP), imazalil (IMZ), propamocarb (PPC), chlorpropham (CPH), boscalid (BOS), azoxystrobin (AZX), prosulfocarb (PSC), tebuconazole (TEB), dimethomorph (DMM), difenoconazole (DFZ), fluazifop (FLP), piperonyl butoxide (PBO), chlorpyrifos (CPF), fluopyram (FPM), linuron (LIN), epoxiconazole (EPX), pyrimethanil (PYM), isoprocarb (ISP), carbaryl (CRB), thiamethoxam (TMX), tebufenozide (TBZ), pirimiphos-methyl (PPM), metamitron (MTT), ethoxyquin (ETQ), fluopicolide (FPC), resmethrin (RMT), iprovalicarb (IPV), cyromazine (CYR), fenhexamid (FHX), fosthiazate (FST), clothianidin (CLT), and imidacloprid (IMI).

**Table 3:** Hazard index values of acute risks for adults and children.

**Acute Risk for Adults**			
**Vegetable**	**HIa Min**	**HIa Max**	**HIa Mean**
Celery	2.96 × 10^−4^	2.19 × 10^−1^	2.87 × 10^−2^
Ginger	4.25 × 10^−4^	5.11 × 10^−1^	2.86 × 10^−1^
Potato	1.93 × 10^−4^	1.23 × 10^−1^	1.30 × 10^−2^
Onion and leek	1.82 × 10^−4^	1.21 × 10^−1^	1.84 × 10^−2^
Radish	1.66 × 10^−4^	9.11 × 10^−2^	2.32 × 10^−2^
Carrot	2.32 × 10^−3^	5.46 × 10^−3^	3.89 × 10^−3^
All root vegetables	1.66 × 10^−4^	5.11 × 10^−1^	5.04 × 10^−2^
**Acute Risk for Children**			
**Vegetable**	**HIa Min**	**HIa Max**	**HIa Mean**
Celery	8.52 × 10^−4^	6.31 × 10^−1^	8.26 × 10^−2^
Ginger	6.23 × 10^−5^	7.47 × 10^−2^	2.41 × 10^−2^
Potato	4.59 × 10^−4^	2.92 × 10^−1^	3.09 × 10^−2^
Onion and leek	3.26 × 10^−4^	2.16 × 10^−1^	3.50 × 10^−2^
Radish	9.72 × 10^−5^	2.83 × 10^−1^	7.14 × 10^−2^
Carrot	7.98 × 10^−3^	1.88 × 10^−2^	1.34 × 10^−2^
All root vegetables	6.23 × 10^−5^	6.31 × 10^−1^	4.49 × 10^−2^

**Table 4:** Hazard index values of chronic risks for adults and children.

**Chronic Risk for Adults**			
**Vegetable**	**HIc Min**	**HIc Max**	**HIc Mean**
Celery	1.96 × 10^−5^	5.90 × 10^−2^	5.73 × 10^−3^
Ginger	7.52 × 10^−5^	3.89 × 10^−2^	1.08 × 10^−2^
Potato	4.68 × 10^−5^	1.51 × 10^−2^	2.47 × 10^−3^
Onion and leek	5.19 × 10^−5^	2.40 × 10^−2^	3.31 × 10^−3^
Radish	4.51 × 10^−5^	3.63 × 10^−3^	7.42 × 10^−4^
Carrot	1.80 × 10^−5^	6.38 × 10^−3^	1.38 × 10^−3^
All root vegetables	1.80 × 10^−5^	5.90 × 10^−2^	4.08 × 10^−3^
**Chronic Risk for Children**			
**Vegetable**	**HIc Min**	**HIc Max**	**HIc Mean**
Celery	3.66 × 10^−5^	1.10 × 10^−1^	1.07 × 10^−2^
Ginger	1.40 × 10^−4^	7.27 × 10^−2^	2.02 × 10^−2^
Potato	8.73 × 10^−5^	2.83 × 10^−2^	4.62 × 10^−3^
Onion and leek	9.68 × 10^−5^	4.48 × 10^−2^	6.18 × 10^−3^
Radish	8.42 × 10^−5^	6.77 × 10^−3^	1.39 × 10^−3^
Carrot	3.36 × 10^−5^	1.19 × 10^−2^	2.57 × 10^−3^
All root vegetables	3.36 × 10^−5^	1.10 × 10^−1^	7.62 × 10^−3^

## Data Availability

The original contributions presented in this study are included in the article/Supplementary Materials. Further inquiries can be directed to the corresponding author(s).

## Supplementary Materials

The following supporting information can be downloaded at: https://www.mdpi.com/article/10.3390/jox15040125/s1, Table S1: Pesticide standards used for analysis of root and tuber vegetables; Table S2: Definition and individual scores of indices for the pesticide residual risk ranking; Table S3: Summary table for LD50, ADI, and ARfD values and assigned scores for indices A and B. The LD50 values are adopted from the WHO database [35], the EPA Fact Sheet [36,37,38], and scientific opinion on hexachlorocyclohexanes [39]. Most of the ADI values are sourced from the EU Pesticide database [40], and the values that did not exist in this database were taken from the JMPR database [42]. ARfD values were sourced from from the EU Pesticide database [40], (EFSA, 2024) [45], (WHO & FAO, 2004) [46], and (EFSA, 2019) [47]; Table S4: The MRL values (mg/kg) were sourced from the EU pesticide database [40]. Empty cells: those pesticides were not detected in those vegetables; Table S5: Assigned scores for indices A-F for the calculation of the total risk score of pesticides in our study; Table S6: Assigned scores for indices A-F for the calculation of the total risk score of pesticides in vegetables from RASFF; Table S7: Descriptive statistics of detected pesticides in vegetables (mg/kg); Table S8: Vegetables positive for pesticide residues, hierarchical cluster analysis; Table S9: HQa (acute hazard quotient) and HQc (chronic hazard quotient) for adults and children for each evaluated pesticide: Table S10: Monte Carlo simulation: 10th and 90th percentiles of HIc.
