# Supplementary material for: Prioritization and Sensitivity of Pesticide Risks from Root and Tuber Vegetables
Source: J Xenobiot. 2025 Aug 3;15(4):125. doi: 10.3390/jox15040125 (PMC12387184; doi:10.3390/jox15040125)
Supplement: Supplementary file 1 [file jox-15-00125-s001.zip › jox-3746640-supplementary.pdf]

Supplementary Material: Prioritization and Sensitivity of Pesticide Risks from Root and Tuber Vegetables

Milica Lučić and Antonije Onjia

Table S1. Pesticide standards used for analysis of root and tuber vegetables.

Pesticide*	Concentration	Solvent	Purity (%)	Standard Name (Catalog #)	Supplier
**Azoxystrobin**	100 mg/L (each)	Acetonitrile	≥ 98	LabStandard® Pesticides Kit (KIT4AB3L160) – Thermo 63899 mix	Lab Instruments
	100 µg/mL (each)	Acetonitrile	≥ 98	LC Multiresidue Pesticide Std. #4 (Cat.# 31975)	Restek
	100 mg neat (solid)	(neat solid)	> 99	Azoxystrobin (DRE-C10413000) – individual CRM	Dr. Ehrenstorfer
**Boscalid**	100 mg/L (each)	Acetonitrile	≥ 98	LabStandard® Pesticides Kit (KIT4AB3L160) – contains Boscalid	Lab Instruments
	100 µg/mL (each)	Acetonitrile	≥ 98	LC Multiresidue Pesticide Std. #4 (Cat.# 31975) – contains Boscalid	Restek
	100 mg neat (solid)	(neat solid)	≥ 98	Boscalid (DRE-C18842500) – neat standard (100 mg)	Dr. Ehrenstorfer
**Carbaryl**	100 mg/L (each)	Acetonitrile	≥ 98	LabStandard® Pesticides Kit (KIT4AB3L160) – contains Carbaryl	Lab Instruments
	100 µg/mL (each)	Acetonitrile	≥ 98	LC Multiresidue Pesticide Std. #1 (Cat.# 31972) – contains Carbaryl	Restek
	100 mg neat (solid)	(neat solid)	≥ 99	Carbaryl (DRE-C10085000) – individual CRM (100 mg)	Dr. Ehrenstorfer
**Chlorpropham**	100 mg/L (each)	Acetonitrile/Toluene	≥ 98	LabStandard® Pesticides Kit (KIT4AB3L160) – contains Chlorpropham	Lab Instruments
	100 mg neat (solid)	(neat solid)	≥ 98	Chlorpropham (CIPC) – individual CRM (100 mg)	Dr. Ehrenstorfer
**Chlorpyrifos**	100 mg/L (each)	Acetonitrile	≥ 98	LabStandard® Pesticides Kit (KIT4AB3L160) – contains Chlorpyrifos	Lab Instruments
	100 µg/mL (each)	Acetonitrile	≥ 98	California Pesticide Std. #1 (Cat.# 34124) – contains Chlorpyrifos	Restek
	100 mg neat (solid)	(neat solid)	≥ 98	Chlorpyrifos (DRE-C13535000) – individual CRM (100 mg)	Dr. Ehrenstorfer
**Clothianidin**	100 mg/L (each)	Methanol	≥ 98	*Polar Pesticides Mix* (Qpp-Lab® kit, 100 mg/L each) – contains Clothianidin	Lab Instruments
	100 µg/mL (each)	Acetonitrile	≥ 98	LC Multiresidue Pesticide Std. #5 (Cat.# 31976) – contains Clothianidin	Restek
	100 µg/mL solution	Methanol	≥ 98	Clothianidin (DRE-C11836500) – individual CRM (50 mg)	Dr. Ehrenstorfer
**Cyromazine**	100 mg/L (each)	Acetonitrile	≥ 98	LabStandard® Pesticides Kit (KIT4AB3L160) – contains Cyromazine	Lab Instruments
	100 µg/mL (each)	Acetonitrile	≥ 98	LC Multiresidue Pesticide Std. #4 (Cat.# 31975) – contains Cyromazine	Restek
	100 mg/L solution	Acetonitrile	≥ 98	Cyromazine (DRE-C10684400) – individual CRM (50 mg)	Dr. Ehrenstorfer
**Difenoconazole**	100 mg/L (each)	Acetonitrile	≥ 98	LabStandard® Pesticides Kit (KIT4AB3L160) – contains Difenoconazole	Lab Instruments

	100 µg/mL (each)	Acetonitrile	≥ 98	LC Multiresidue Pesticide Std. #6 (Cat.# 31977) – contains Difenoconazole	Restek
	100 mg neat (solid)	(neat solid)	> 99	Difenoconazole (DRE-C11944600) – individual CRM (50 mg)	Dr. Ehrenstorfer
**Dimethomorph**	100 mg/L (each)	Acetonitrile	≥ 98	LabStandard® Pesticides Kit (KIT4AB3L160) – contains Dimethomorph	Lab Instruments
	100 µg/mL (each)	Acetonitrile	≥ 98	LC Multiresidue Pesticide Std. #1 (Cat.# 31972) – contains Dimethomorph	Restek
	100 mg neat (solid)	(neat solid)	≥ 98	Dimethomorph (DRE-C11048800) – individual CRM (50 mg)	Dr. Ehrenstorfer
**Epoxiconazole**	100 mg/L (each)	Acetonitrile	≥ 98	LabStandard® Pesticides Kit (KIT4AB3L160) – contains Epoxiconazole	Lab Instruments
	100 µg/mL (each)	Acetonitrile	≥ 98	LC Multiresidue Pesticide Std. #6 (Cat.# 31977) – contains Epoxiconazole	Restek
	100 mg neat (solid)	(neat solid)	≥ 98	Epoxiconazole (DRE-C13385500) – individual CRM (50 mg)	Dr. Ehrenstorfer
**Ethoxyquin**	100 mg/L (each)	Acetonitrile	≥ 95	LabStandard® Pesticides Kit (KIT4AB3L160) – contains Ethoxyquin	Lab Instruments
	100 mg neat (solid)	(neat solid)	≥ 95	Ethoxyquin (DRE-C00009153) – individual CRM (100 mg)	Dr. Ehrenstorfer
**Fenhexamid**	100 mg/L (each)	Acetonitrile	≥ 98	LabStandard® Pesticides Kit (KIT4AB3L160) – contains Fenhexamid	Lab Instruments
	100 µg/mL (each)	Acetonitrile	≥ 98	LC Multiresidue Pesticide Std. #4 (Cat.# 31975) – contains Fenhexamid	Restek
	100 mg neat (solid)	(neat solid)	≥ 98	Fenhexamid (DRE-C12683300) – individual CRM (50 mg)	Dr. Ehrenstorfer
**Fluazifop**	100 mg/L (each)	Acetonitrile	≥ 98	LabStandard® Pesticides Kit (KIT4CH3L676) – contains Fluazifop	Lab Instruments
	100 mg neat (solid)	(neat solid)	≥ 98	Fluazifop-P-butyl (DRE-C07924146) – individual CRM (100 mg)	Dr. Ehrenstorfer
**Fluopicolide**	100 mg/L (each)	Acetonitrile	≥ 98	LabStandard® Pesticides Kit (KIT4CH3L676) – contains Fluopicolide	Lab Instruments
	100 mg neat (solid)	(neat solid)	≥ 98	Fluopicolide (DRE-C23911000) – individual CRM (50 mg)	Dr. Ehrenstorfer
**Fluopyram**	100 mg/L (each)	Acetonitrile	≥ 98	LabStandard® Pesticides Kit (KIT4CH3L676) – contains Fluopyram	Lab Instruments
	100 mg neat (solid)	(neat solid)	≥ 98	Fluopyram (DRE-C65806600) – individual CRM (50 mg)	Dr. Ehrenstorfer
**Fosthiazate**	100 mg/L (each)	Acetonitrile	≥ 98	LabStandard® Pesticides Kit (KIT4CH3L676) – contains Fosthiazate	Lab Instruments
	100 mg neat (solid)	(neat solid)	≥ 98	Fosthiazate (DRE-C09888600) – individual CRM (50 mg)	Dr. Ehrenstorfer
**Imazalil**	100 mg/L (each)	Acetonitrile	≥ 98	LabStandard® Pesticides Kit (KIT4AB3L160) – contains Imazalil	Lab Instruments
	100 µg/mL (each)	Acetonitrile	≥ 98	LC Multiresidue Pesticide Std. #4 (Cat.# 31975) – contains Imazalil	Restek
	100 mg neat (solid)	(neat solid)	≥ 98	Imazalil (DRE-C35554000) – individual CRM (100 mg)	Dr. Ehrenstorfer
**Imidacloprid**	100 mg/L (each)	Acetonitrile	≥ 98	LabStandard® Pesticides Kit (KIT4CH3L676) – contains Imidacloprid	Lab Instruments
	100 µg/mL (each)	Acetonitrile	≥ 98	LC Multiresidue Pesticide Std. #4 (Cat.# 31975) – contains Imidacloprid	Restek

	100 mg neat (solid)	(neat solid)	≥ 99	Imidacloprid (DRE-C13826100) – individual CRM (50 mg)	Dr. Ehrenstorfer
**Iprovalicarb**	100 mg/L (each)	Acetonitrile	≥ 98	LabStandard® Pesticides Kit (KIT4CH3L676) – contains Iprovalicarb	Lab Instruments
	100 µg/mL (each)	Acetonitrile	≥ 98	LC Multiresidue Pesticide Std. #3 (Cat.# 31974) – contains Iprovalicarb	Restek
	100 mg neat (solid)	(neat solid)	≥ 98	Iprovalicarb (DRE-C14092300) – individual CRM (50 mg)	Dr. Ehrenstorfer
**Isoprocarb**	100 mg/L (each)	Acetonitrile	≥ 98	LabStandard® Pesticides Kit (KIT4CH3L676) – contains Isoprocarb	Lab Instruments
	100 µg/mL (each)	Acetonitrile	≥ 98	LC Multiresidue Pesticide Std. #3 (Cat.# 31974) – contains Isoprocarb	Restek
	100 mg neat (solid)	(neat solid)	≥ 98	Isoprocarb (DRE-C00263140) – individual CRM (100 mg)	Dr. Ehrenstorfer
**Linuron**	100 mg/L (each)	Acetonitrile	≥ 98	LabStandard® Pesticides Kit (KIT4CH3L676) – contains Linuron	Lab Instruments
	100 µg/mL (each)	Acetonitrile	≥ 98	LC Multiresidue Pesticide Std. #3 (Cat.# 31974) – contains Linuron	Restek
	100 mg neat (solid)	(neat solid)	≥ 98	Linuron (DRE-C33055000) – individual CRM (100 mg)	Dr. Ehrenstorfer
**Metamitron**	100 mg/L (each)	Acetonitrile	≥ 98	LabStandard® Pesticides Kit (KIT4CH3L676) – contains Metamitron	Lab Instruments
	100 mg neat (solid)	(neat solid)	≥ 98	Metamitron (DRE-C04139405) – individual CRM (100 mg)	Dr. Ehrenstorfer
**Metolachlor**	100 mg/L (each)	Acetonitrile	≥ 98	LabStandard® Pesticides Kit (KIT4CH3L676) – contains Metolachlor	Lab Instruments
	100 mg neat (solid)	(neat solid)	≥ 98	Metolachlor (DRE-C51218000) – individual CRM (100 mg)	Dr. Ehrenstorfer
**Piperonyl butoxide**	100 mg/L (each)	Acetonitrile	≥ 98	LabStandard® Pesticides Kit (KIT4AB3L160) – contains Piperonyl butoxide	Lab Instruments
	100 µg/mL (each)	Acetonitrile	≥ 98	LC Multiresidue Pesticide Std. #4 (Cat.# 31975) – contains Piperonyl butoxide	Restek
	100 mg neat (solid)	(neat solid)	≥ 98	Piperonyl butoxide (DRE-C00005103) – individual CRM (100 mg)	Dr. Ehrenstorfer
**Pirimiphos-methyl**	100 mg/L (each)	Acetonitrile	≥ 98	LabStandard® Pesticides Kit (KIT4CH3L676) – contains Pirimiphos-methyl	Lab Instruments
	100 mg neat (solid)	(neat solid)	≥ 98	Pirimiphos-methyl (DRE-C29232930) – individual CRM (50 mg)	Dr. Ehrenstorfer
**Propamocarb**	100 mg/L (each)	Methanol	≥ 98	LabStandard® *Polar Pesticides Mix* (Qpp-Lab) – contains Propamocarb	Lab Instruments
	100 µg/mL (each)	Acetonitrile	≥ 98	LC Multiresidue Pesticide Std. #9 (Cat.# 31980) – contains Propamocarb (free base)	Restek
	100 mg neat (solid)	(neat solid)	≥ 98	Propamocarb HCl (DRE-C24579HCL) – individual CRM (100 mg)	Dr. Ehrenstorfer
**Prosulfocarb**	100 mg/L (each)	Acetonitrile	≥ 98	LabStandard® Pesticides Kit (KIT4CH3L676) – contains Prosulfocarb	Lab Instruments
	100 mg neat (solid)	(neat solid)	≥ 98	Prosulfocarb (DRE-C52888800) – individual CRM (100 mg)	Dr. Ehrenstorfer
**Pyrimethanil**	100 mg/L (each)	Acetonitrile	≥ 98	LabStandard® Pesticides Kit (KIT4AB3L160) – contains Pyrimethanil	Lab Instruments
	100 µg/mL (each)	Acetonitrile	≥ 98	LC Multiresidue Pesticide Std. #4 (Cat.# 31975) – contains Pyrimethanil	Restek

	100 mg neat (solid)	(neat solid)	≥ 98	Pyrimethanil (DRE-C53112280) – individual CRM (50 mg)	Dr. Ehrenstorfer
**Resmethrin**	100 mg/L (each)	Acetonitrile	> 99	LabStandard® Pesticides Kit (KIT4CH3L676) – contains Resmethrin	Lab Instruments
	100 mg neat (solid)	(neat solid)	> 99	Resmethrin (DRE-C10453860) – individual CRM (100 mg)	Dr. Ehrenstorfer
**Tebuconazole**	100 mg/L (each)	Acetonitrile	≥ 98	LabStandard® Pesticides Kit (KIT4CH3L676) – contains Tebuconazole	Lab Instruments
	100 µg/mL (each)	Acetonitrile	≥ 98	LC Multiresidue Pesticide Std. #6 (Cat.# 31977) – contains Tebuconazole	Restek
	100 mg neat (solid)	(neat solid)	≥ 98	Tebuconazole (DRE-C10753400) – individual CRM (50 mg)	Dr. Ehrenstorfer
**Tebufenozide**	100 mg/L (each)	Acetonitrile	≥ 98	LabStandard® Pesticides Kit (KIT4AB3L160) – contains Tebufenozide	Lab Instruments
	100 µg/mL (each)	Acetonitrile	≥ 98	LC Multiresidue Pesticide Std. #4 (Cat.# 31975) – contains Tebufenozide	Restek
	100 mg neat (solid)	(neat solid)	≥ 98	Tebufenozide (DRE-C11241000) – individual CRM (50 mg)	Dr. Ehrenstorfer
**Tebufenpyrad**	100 mg/L (each)	Acetonitrile	≥ 98	LabStandard® Pesticides Kit (KIT4AB3L160) – contains Tebufenpyrad	Lab Instruments
	100 µg/mL (each)	Acetonitrile	≥ 98	LC Multiresidue Pesticide Std. #4 (Cat.# 31975) – contains Tebufenpyrad	Restek
	100 mg neat (solid)	(neat solid)	≥ 98	Tebufenpyrad (DRE-C11916800) – individual CRM (50 mg)	Dr. Ehrenstorfer
**Thiamethoxam**	100 mg/L (each)	Methanol	≥ 98	LabStandard® *Polar Pesticides Mix* (Qpp-Lab) – contains Thiamethoxam	Lab Instruments
	100 µg/mL (each)	Acetonitrile	≥ 98	LC Multiresidue Pesticide Std. #5 (Cat.# 31976) – contains Thiamethoxam	Restek
	100 mg neat (solid)	(neat solid)	≥ 98	Thiamethoxam (DRE-C15371900) – individual CRM (50 mg)	Dr. Ehrenstorfer

* *Only detected pesticides are listed.*

Table S2. Definition and individual scores of indices for the pesticide residual risk ranking.

Index	Item	Definition	Score	Definition	Score	Definition	Score	Definition	Score
A	Toxicity (mg/kg)	Low	2	Moderate	3	High	4	Hypertoxic	5
B	Potency (mg/kg)	$>10^{-2}$	0	$>10^{-4}$–10^{-2}	1	$>10^{-6}$–10^{-4}	2	$<10^{-6}$	3
C	Proportion of diet (%)	<2.5	0	2.5–20	1	20–50	2	50–100	3
D	Frequency of dosing (%)	<2.5	0	2.5–20	1	20–50	2	50–100	3
E	Evidence for high exposure groups	No	0	Unlikely	1	Likely	2	Existing	3
F	Residue level (mg/kg)	ND	1	<MRL	2	≥1–10 MRL	3	≥10 MRL	4

Table S3. Summary table for LD_{50}, ADI, and ARfD values and assigned scores for indices A and B. The LD_{50} values are adopted from the WHO database [35], the EPA Fact Sheet [36–38], and scientific opinion on hexachlorocyclohexanes [39]. Most of the ADI values are sourced from the EU Pesticide database [40], and the values that did not exist in this database were taken from the JMPR database [42]. ARfD values were sourced from the EU Pesticide database [40], (EFSA, 2024) [45], (WHO & FAO, 2004) [46], and (EFSA, 2019) [47].

**No**	**Pesticide**	**LD_{50} (WHO, 2020)**	**A**	**ADI - EU Pesticide database** [40]	**B**	**ARfD - EU Pesticide database** [40]
		(mg/kg)		(mg/kg/day)		mg/kg bw
		Pesticides detected in this study				
1	Pyrimethanil	4159	2	0.17	0	1 [45]
2	Boscalid	>5000	2	0.04	0	Not Applicable
3	Tebuconazole	1700	3	0.03	0	0.03
4	Metolachlor	2780	2	Not Applicable	3	0.5 [46]
5	Azoxystrobin	>5000	2	0.2	0	Not Applicable
6	Fluopyram	>2000	2	0.012	0	0.5
7	Difenoconazole	1453	3	0.01	1	0.16
8	Prosulfocarb	1820	3	0.005	1	0.1
9	Linuron	4000	2	0.003	1	Not Applicable
10	Carbaryl	c300	3	0.0075	1	0.01
11	Resmethrin	2000	2	0.03	0	Not Applicable
12	Tebufenpyrad	595	3	0.01	1	0.02
13	Tebufenozide	>5000	2	0.02	0	Not Applicable
14	Iprovalicarb	>5000	2	0.015	0	Not Applicable
15	Pirimiphos-methyl	1667	3	0.004	1	0.15
16	Chlorpropham	>5000	2	0.05	0	0.5
17	Clothianidin	389	3	0.097	0	0.1
18	Fluopicolide	>5000	2	0.08	0	0.18
19	Fosthiazate	73* [38]	3	0.004	1	0.005
20	Fluazifop	2451	2	0.01	1	0.017
21	Epoxiconazole	>2000* [36]	2	0.008	1	0.023
22	Dimethomorph	3500	2	0.05	0	0.6
23	Thiamethoxam	871	3	0.026	0	0.5
24	Piperonyl butoxide	>7500	2	0.2 [42]	0	Not applicable
25	Imidacloprid	450	3	0.06	0	0.08
26	Cyromazine	3300	2	0.06	0	0.1
27	Fenhexamid	>5000	2	0.2	0	Not Applicable
28	Ethoxyquin	>2000* [37]	2	Not Applicable	3	Not applicable
29	Imazalil	227	3	0.025	0	0.05
30	Chlorpyrifos	135	3	Not set	3	0.005 [47]
31	Metamitron	1182	3	0.03	0	0.1
32	Propamocarb	8600	2	0.29	0	1
33	Isoprocarb	403	3	Not Applicable	3	Not applicable
		Pesticides reported in RASFF				
1	Pirimiphos-methyl	1667	3	0.004	1	
2	Linuron	4000	2	0.003	1	
3	Dieldrin	prohibited	5	Not Applicable	3	
4	Chlorpyriphos	135	3	Not set due to insufficient data #	3	
5	Oxamyl	2.50	5	0.0001	2	
6	Fenamiphos	15	4	0.0008	1	
7	Propiconazole	1520	3	0.04	0	

8	Diazinon	300	3	0.0002	1
9	Quintozene	>10000	2	0.01	1
10	Ethoprophos	D26	5	Not set due to concerns	3
11	Bromide	/	2	Not Applicable	3
12	Fosthiazate	73*	3	0.004	1
13	Tebuconazole	1700	3	0.03	0
14	Ethylene oxide	prohibited	5	Not assessed	3
15	Diafenthiuron	2068	2	Not Applicable	3
16	Thiamethoxam	871	3	0.026	0
17	Carbendazim	>10000	2	0.02	0
18	Chlorantraniliprole	>5000	2	1.56	0
19	Cypermethrin	c250	3	0.005	1
20	Emamectin	53-237	3	0.0005	1
21	Fipronil	92	3	0.0002	1
22	Indoxacarb	268	3	0.005	1
23	Iprodione	3500	2	0.02	0
24	Lufenuron	>2000	2	0.015	0
25	Acetamiprid	c140	3	0.005	1
26	Bifenthrin	c55	3	0.015	0
27	Chlorfenapyr	441	3	0.015	0
28	Imazalil	227	3	0.025	0
29	Flutolanil	>10000	2	0.09	0
30	Fluazifop	2451	2	0.01	1
31	Imidacloprid	450	3	0.06 #	0
32	Omethoate	50	4	Not Applicable	3
33	Lambda-cyhalothrin	c56	3	0.0025	1
34	Malathion	c2100	2	0.03	0
35	Thiabendazole	3330	2	0.1	0
36	Flonicamid	884	3	0.025	0
37	Chlormequat	433	3	0.04	0
38	Thiophanate-methyl	>5000	2	0.02	0
39	Prochloraz	1600	3	0.01	1
40	Monocrotophos	14	4	0.0006	1
41	Griseofulvin	/	3	Nema	3
42	Carbofuran	8	4	0.00015	1
43	Metalaxyl	670	3	0.08	0
44	HCH beta (Alexander et al.. 2005)	8.0–15.0	4	Not Applicable	3
45	HCH (Alexander et al., 2005)	0.5–5	5	Not Applicable	3
46	HCH gamma (Alexander et al.. 2005)	125	3	Not Applicable	3

Table S4. The MRL values (mg/kg) were sourced from the EU pesticide database [40]. Empty cells: those pesticides were not detected in those vegetables.

Pesticides	Celery	Onion	Potato	Ginger	Carrot	Radish	Horseradish	Spring onion	Leek
Pyrimethanil	0.05	0.2	0.05	*	*	*	*	*	*
Boscalid	0.9	5	*	*	2	*	*	6	9
Tebuconazole	0.5	*	*	*	0.4	*	*	*	0.6
Metolachlor	0.05	*	0.05	0.05	*	*	*	*	*
Azoxystrobin	0.3	10	7	*	1	*	*	*	*
Fluopyram	0.05	*	0.08	*	*	*	*	*	*
Difenoconazole	0.3	*	*	*	*	*	*	*	*
Prosulfocarb	0.3	*	*	*	1	*	*	*	*
Linuron	0.05	*	*	*	*	*	*	*	*
Carbaryl	0.05	0.02	0.01	*	*	*	*	*	*
Resmethrin	0.05	0.01	0.01	*	*	*	*	*	*
Tebufenpyrad	*	*	*	0.01	*	*	*	*	*
Tebufenozide	*	*	*	10	*	*	*	*	*
Iprovalicarb	*	*	*	0.01	*	*	*	*	*
Pirimiphos-methyl	*	*	*	0.01	*	*	*	*	*
Chlorpropham	*	*	0.35	*	*	*	*	*	*
Clothianidin	*	*	0.01	*	*	*	*	*	*
Fluopicolide	*	1	0.03	*	*	*	*	*	*
Fosthiazate	0.02	*	0.02	*	*	*	*	*	*
Fluazifop	*	*	0.15	*	*	*	*	*	*
Epoxiconazole	*	*	0.01	*	*	*	*	*	*
Dimethomorph	*	0.6	0.05	*	*	*	*	*	*
Thiamethoxam	*	*	0.01	0.02	*	0.01	*	*	0.1
Piperonyl butoxide	*	*	2	*	*	*	*	*	*
Imidacloprid	*	0.01	0.01	0.02	*	*	*	*	*
Cyromazine	*	*	0.01	*	*	*	*	*	*
Fenhexamid	*	3	*	*	*	*	*	*	*
Ethoxyquin	*	0.05	*	*	*	*	*	*	*
Imazalil	*	0.01	0.01	*	*	*	*	*	*
Chlorpyrifos	0.01	0.01	0.01	0.02	0.01	0.01	*	*	*
Metamitron	*	*	*	*	*	*	0.01	*	*
Propamocarb	*	*	*	*	*	3	*	*	*
Isoprocarb	0.01	0.01	0.01	0.01	0.01	0.01	0.01	0.01	0.01
Dieldrin	*	*	*	*	0.01	*	*	*	*
Oxamyl	*	*	0.001	*	0.001	*	*	*	*
Fenamiphos	*	*	*	*	0.01	*	*	*	*
Propiconazole	*	*	*	*	0.01	*	*	*	*
Diazinon	*	*	*	*	0.01	*	*	*	*
Quintozene	*	*	*	*	0.01	*	*	*	*
Ethoprophos	*	*	*	*	0.01	*	*	*	*
Bromide	30	*	*	*	0.01	*	*	*	*
Ethylene oxide	0.02	*	*	0.01	*	0.1	*	*	*
Diafenthiuron	*	*	*	*	*	*	*	*	0.01
Carbendazim	*	*	0.1	0.1	*	*	*	*	0.1
Chlorantraniliprole	*	0.01	*	*	*	*	*	*	*
Cypermethrin	*	0.1	*	*	*	*	*	*	*
Emamectin	*	0.002	*	*	*	*	*	*	*

Fipronil	*	0.005	*	0.01	*	*	*	*	*
Indoxacarb	*	0.01	*	*	*	*	*	*	*
Iprodione	*	0.01	*	*	*	*	*	*	*
Lufenuron	*	0.01	*	*	*	*	*	*	*
Acetamiprid	*	0.02	*	*	*	*	*	*	*
Bifenthrin	*	0.01	*	*	*	*	*	*	*
Chlorfenapyr	*	0.02	*	*	*	*	*	*	*
Flutolanil	*	*	0.1	*	*	*	*	*	*
Omethoate	*	*	0.01	*	*	*	*	*	*
Lambda-cyhalothrin	*	*	0.01	*	*	*	*	*	*
Malathion	*	*	0.02	*	*	*	*	*	*
Thiabendazole	*	*	0.04	*	*	*	*	*	*
Flonicamid	*	*	0.2	*	*	*	*	*	*
Chlormequat	*	*	0.01	*	*	*	*	*	*
Thiophanate-methyl	*	*	0.1	*	*	*	*	*	*
Prochloraz	*	*	0.03	*	*	*	*	*	*
Monocrotophos	*	*	0.01	*	*	*	*	*	0.01
Griseofulvin	*	*	*	0.02	*	*	*	*	*
Carbofuran	*	*	*	0.004	*	*	*	*	*
Metalaxyl	*	*	*	0.05	*	*	*	*	*
HCH beta	*	*	*	0.01	*	*	*	*	*
HCH	*	*	*	0.01	*	*	*	*	*
HCH gamma	*	*	*	0.01	*	*	*	*	*

Table S5. Assigned scores for indices A-F for the calculation of the total risk score of pesticides in our study.

	Toxicity score LD50	Potency score ADI	Proportion of diet (%) score	Frequency of dosing (%) score	Evidence for high exposure groups score	Residue level score	Total score
Pesticide	A	B	C	D	E	F	S
Pyrimethanil	2	0	0/1*	1	3	1.058	10.6
Boscalid	2	0	0	1	3	1.116	8.9
Tebuconazole	3	0	0	1	3	1.047	12.6
Metolachlor	2	3	0/1*	1	3	1.105	27.6
Azoxystrobin	2	0	0/1*	1	3	1.174	11.7
Fluopyram	2	0	0/1*	1	3	1.105	11.0
Difenoconazole	3	1	0	1	3	1.070	17.1
Prosulfocarb	3	1	0	1	3	1.070	17.1
Linuron	2	1	0	1	3	1.035	12.4
Carbaryl	3	1	0	1	3	1.012	16.2
Resmethrin	2	0	0	1	3	1.012	8.1
Tebufenpyrad	3	1	0	1	3	1.198	19.2
Tebufenozide	2	0	0	1	3	1.047	8.4
Iprovalicarb	2	0	0	1	3	1.023	8.2
Pirimiphos-methyl	3	1	0	1	3	1.023	16.4
Chlorpropham	2	0	1	1	3	1.105	11.0
Clothianidin	3	0	1	1	3	1.070	16.0
Fluopicolide	2	0	0/1*	1	3	1.023	11.0
Fosthiazate	3	1	1	1	3	1.035	16.0
Fluazifop	2	1	1	1	3	1.012	10.2
Epoxiconazole	2	1	1	1	3	1.047	20.7
Dimethomorph	2	0	0/1*	1	3	1.035	15.2
Thiamethoxam	3	0	0/1*	1	3	1.047	15.7
Piperonyl butoxide	2	0	0/1*	1	3	1.023	10.2
Imidacloprid	3	0	0/1*	1	3	1.047	15.7
Cyromazine	2	0	1	1	3	1.023	10.2
Fenhexamid	2	0	0	1	3	1.012	8.1
Ethoxyquin	2	3	0	1	3	1.012	20.2
Imazalil	3	0	0	1	3	1.035	12.4
Chlorpyrifos	3	3	0	1	3	1.058	25.4
Metamitron	3	0	0	1	3	1.023	12.3
Propamocarb	2	0	0/1*	1	3	1.058	10.6
Isoprocarb	3	3	0	1	3	1.023	24.6

*for potato score C was 1

Table S6. Assigned scores for indices A-F for the calculation of the total risk score of pesticides in vegetables from RASFF.

	Toxicity score LD50	Potency score ADI	Proportion of diet (%) score	Frequency of dosing (%) score	Evidence for high exposure groups score	Residue level score	Total score
Pesticide	A	B	C	D	E	F	S
Pirimiphos-methyl	3	1	0	1	3	1.023	16.4
Linuron	2	1	0	1	3	1.214	14.6
Dieldrin	5	3	0	1	3	1.038	33.2
Chlorpyriphos	3	3	0/1*	1	3	1.344	40.3
Oxamyl	5	2	0/1*	1	3	1.160	40.6
Fenamiphos	4	1	0	1	3	1.137	22.7
Propiconazole	3	0	0	1	3	1.015	12.2
Diazinon	3	1	0	1	3	1.023	16.4
Quintozene	2	1	0	1	3	1.015	12.2
Ethoprophos	5	3	0	1	3	1.023	32.7
Bromide	2	3	0	1	3	1.023	20.5
Fosthiazate	3	1	0/1*	1	3	1.099	22.0
Tebuconazole	3	0	0	1	3	1.015	12.2
Ethylene oxide	5	3	0	1	3	1.427	45.7
Diafenthiuron	2	3	0	1	3	1.031	20.6
Thiamethoxam	3	0	0	1	3	1.038	12.5
Carbendazim	2	0	0/1*	1	3	1.092	10.9
Chlorantraniliprole	2	0	0	1	3	1.023	8.2
Cypermethrin	3	1	0	1	3	1.015	16.2
Emamectin	3	1	0	1	3	1.015	16.2
Fipronil	3	1	0	1	3	1.053	16.9
Indoxacarb	3	1	0	1	3	1.023	16.4
Iprodione	2	0	0	1	3	1.023	8.2
Lufenuron	2	0	0	1	3	1.023	8.2
Acetamiprid	3	1	0	1	3	1.023	16.4
Bifenthrin	3	0	0	1	3	1.023	12.3
Chlorfenapyr	3	0	0	1	3	1.015	12.2
Imazalil	3	0	0/1*	1	3	1.092	16.4
Flutolanil	2	0	0/1*	1	3	1.015	10.2
Fluazifop	2	1	0/1*	1	3	1.015	15.2
Imidacloprid	3	0	0/1*	1	3	1.092	16.4
Omethoate	4	3	0/1*	1	3	1.015	35.5
Lambda-cyhalothrin	3	1	0/1*	1	3	1.023	20.5
Malathion	2	0	0/1*	1	3	1.023	10.2
Thiabendazole	2	0	0/1*	1	3	1.023	10.2
Flonicamid	3	0	0/1*	1	3	1.008	15.1
Chlormequat	3	0	0/1*	1	3	1.023	15.3
Thiophanate-methyl	2	0	0/1*	1	3	1.038	10.4
Prochloraz	3	1	0/1*	1	3	1.023	20.5
Monocrotophos	4	1	0	1	3	1.023	20.5
Griseofulvin	3	3	0	1	3	1.015	24.4
Carbofuran	4	1	0	1	3	1.015	20.3
Metalaxyl	3	0	0	1	3	1.015	12.2
HCH beta	4	3	0	1	3	1.015	28.4

HCH	5	3	0	1	3	1.015	32.5
HCH gamma	3	3	0	1	3	1.015	24.4

*for potato score C was 1

Table S7. Descriptive statistics of detected pesticides in vegetables (mg/kg).

Pesticide	Min	Max	Mean	Median
Pyrimethanil	0.014	0.040	0.024	0.023
Boscalid	0.010	0.57	0.132	0.051
Tebuconazole	0.012	0.32	0.111	0.056
Metolachlor	0.020	9.8	2.45	0.023
Azoxystrobin	0.011	0.55	0.070	0.022
Fluopyram	0.016	0.082	0.040	0.033
Difenoconazole	0.018	0.12	0.065	0.059
Prosulfocarb	0.011	0.41	0.122	0.050
Linuron	0.038	0.097	0.068	0.068
Carbaryl	0.036	0.036	0.036	0.036
Resmethrin	0.022	0.022	0.022	0.022
Tebufenpyrad	0.030	1.1	0.406	0.070
Tebufenozide	0.020	0.030	0.025	0.024
Iprovalicarb	0.020	0.020	0.020	0.020
Pirimiphos-methyl	0.030	0.030	0.030	0.030
Chlorpropham	0.016	0.67	0.150	0.059
Clothianidin	0.012	0.016	0.013	0.012
Fluopicolide	0.020	0.024	0.022	0.022
Fosthiazate	0.011	0.017	0.014	0.013
Fluazifop	0.130	0.13	0.130	0.130
Epoxiconazole	0.023	0.046	0.035	0.035
Dimethomorph	0.016	0.30	0.114	0.027
Thiamethoxam	0.018	0.033	0.026	0.026
Piperonyl butoxide	0.055	0.11	0.083	0.083
Imidacloprid	0.014	0.020	0.017	0.017
Cyromazine	0.020	0.020	0.020	0.020
Fenhexamid	0.020	0.020	0.020	0.020
Ethoxyquin	0.027	0.027	0.027	0.027
Imazalil	0.97	0.97	0.970	0.970
Chlorpyrifos	0.015	0.11	0.063	0.063
Metamitron	0.030	0.030	0.030	0.030
Propamocarb	0.012	0.80	0.200	0.040
Isoprocarb	0.039	0.039	0.039	0.039

Table S8. Vegetables positive for pesticide residues, hierarchical cluster analysis.

Sample	Number	Sample	Number
Cluster A		*Cluster D*	
Celery	1	celery	7
Onion	63	celery	8
Potato	35	onion	66
Potato	36	*Cluster E*	
Potato	37	celery	3
Potato	40	celery	4
Potato	41	celery	15
Potato	42	carrot	81
Cluster B		celery	12
Celery	5	potato	52
Celery	11	carrot	84
Potato	39	leek	74
Onion	69	radish	79
Celery	20	celery	16
Celery	17	carrot	82
Celery	18	ginger	22
Celery	19	potato	60
Celery	6	potato	49
Celery	14	potato	50
Cluster C		carrot	86
Celery	2	radish	80
Celery	9	radish	78
Potato	31	potato	33
Carrot	83	potato	56
Horseradish	75	ginger	23
Onion	65	ginger	24
Onion	62	potato	46
Potato	59	potato	47
Potato	43	onion	67
Ginger	27	radish	76
Celery	10	onion	68
Celery	21	carrot	85
Onion	70	celery	13
Onion	71	leek	72
Potato	51	potato	34
Radish	77	potato	32
Ginger	25	potato	58
Ginger	26	potato	55
Ginger	28	potato	57
Ginger	30	potato	48
Potato	44		
Potato	45		
Potato	54		
Leek	73		
Potato	53		
Onion	64		
Potato	38		

Onion	61
Ginger	29

Table S9. HQa (acute hazard quotient) and HQc (chronic hazard quotient) for adults and children for each evaluated pesticide.

Pesticide	HQc mean adults	HQc mean children		HQc mean adults	HQa mean children
Pyrimethanil	5.32×10^{-6}	9.92×10^{-6}	Pyrimethanil	1.43×10^{-5}	3.44×10^{-5}
Boscalid	1.85×10^{-4}	3.45×10^{-4}	Tebuconazole	1.64×10^{-3}	4.73×10^{-3}
Tebuconazole	5.98×10^{-5}	1.12×10^{-4}	Metolachlor	2.64×10^{-3}	7.59×10^{-3}
Metolachlor	4.68×10^{-4}	8.74×10^{-4}	Fluopyram	6.33×10^{-5}	1.71×10^{-4}
Azoxystrobin	2.05×10^{-5}	3.82×10^{-5}	Difenoconazole	2.61×10^{-4}	7.51×10^{-4}
Fluopyram	1.21×10^{-4}	2.26×10^{-4}	Prosulfocarb	6.63×10^{-4}	1.92×10^{-3}
Difenoconazole	1.48×10^{-4}	2.76×10^{-4}	Carbaryl	3.87×10^{-4}	1.12×10^{-3}
Prosulfocarb	4.63×10^{-4}	8.63×10^{-4}	Tebufenpyrad	1.53×10^{-2}	2.24×10^{-3}
Linuron	1.71×10^{-4}	3.20×10^{-4}	Pirimiphos-methyl	2.15×10^{-5}	3.15×10^{-6}
Carbaryl	1.83×10^{-5}	3.41×10^{-5}	Chlorpropham	4.48×10^{-4}	1.06×10^{-3}
Resmethrin	2.79×10^{-6}	5.21×10^{-6}	Clothianidin	7.48×10^{-5}	1.78×10^{-4}
Tebufenpyrad	1.08×10^{-3}	2.02×10^{-3}	Fluopicolide	3.28×10^{-5}	7.33×10^{-5}
Tebufenozide	1.86×10^{-5}	3.48×10^{-5}	Fosthiazate	1.53×10^{-3}	3.64×10^{-3}
Iprovalicarb	5.07×10^{-6}	9.47×10^{-6}	Fluazifop	1.43×10^{-3}	3.40×10^{-3}
Pirimiphos-methyl	2.85×10^{-5}	5.33×10^{-5}	Epoxiconazole	5.61×10^{-4}	1.33×10^{-3}
Chlorpropham	3.15×10^{-4}	5.87×10^{-4}	Dimethomorph	4.87×10^{-5}	9.50×10^{-5}
Clothianidin	5.42×10^{-6}	1.01×10^{-5}	Thiamethoxam	5.49×10^{-5}	1.36×10^{-4}
Fluopicolide	5.45×10^{-6}	1.02×10^{-5}	Imidacloprid	5.04×10^{-5}	1.09×10^{-4}
Fosthiazate	1.35×10^{-4}	2.51×10^{-4}	Cyromazine	3.74×10^{-5}	8.89×10^{-5}
Fluazifop	1.71×10^{-4}	3.19×10^{-4}	Imazalil	1.37×10^{-3}	2.45×10^{-3}
Epoxiconazole	1.13×10^{-4}	2.12×10^{-4}	Chlorpyrifos	1.27×10^{-3}	3.67×10^{-3}
Dimethomorph	4.75×10^{-5}	8.86×10^{-5}	Metamitron	1.19×10^{-5}	1.13×10^{-6}
Piperonyl butoxide	2.51×10^{-6}	4.69×10^{-6}	Propamocarb	7.02×10^{-5}	1.40×10^{-4}
Thiamethoxam	6.83×10^{-5}	1.28×10^{-4}			
Imidacloprid	5.08×10^{-6}	9.47×10^{-6}			
Cyromazine	4.38×10^{-6}	8.18×10^{-6}			
Fenhexamid	6.03×10^{-7}	1.13×10^{-6}			
Ethoxyquin	3.26×10^{-5}	6.08×10^{-5}			
Imazalil	2.34×10^{-4}	4.37×10^{-4}			
Chlorpyrifos	5.09×10^{-5}	9.50×10^{-5}			
Metamitron	3.80×10^{-6}	7.10×10^{-6}			
Propamocarb	2.02×10^{-5}	3.78×10^{-5}			
Isoprocarb	7.42×10^{-5}	1.38×10^{-4}			

Table S10. Monte Carlo simulation: 10th and 90th percentiles of HIc.

	**10%**	**90%**
All vegetables_Adults	3.60×10^{-3}	4.68×10^{-3}
All vegetables_Children	6.71×10^{-3}	8.73×10^{-3}
Carrot_adults	1.20×10^{-3}	1.59×10^{-3}
Celery_adults	5.01×10^{-3}	6.61×10^{-3}
Ginger_adults	9.47×10^{-3}	1.25×10^{-2}
Onion and Leek_adults	2.89×10^{-3}	3.82×10^{-3}
Potato_adults	2.17×10^{-3}	2.85×10^{-3}
Radish_adults	6.48×10^{-4}	8.54×10^{-4}
Carrot_children	2.25×10^{-3}	2.97×10^{-3}
Celery_children	9.34×10^{-3}	1.23×10^{-2}
Ginger_children	1.77×10^{-2}	2.33×10^{-2}
Onion and Leek_children	5.40×10^{-3}	7.12×10^{-3}
Potato_children	4.04×10^{-3}	5.32×10^{-3}
Radish_children	1.21×10^{-3}	1.60×10^{-3}
